# Dual miRNases for Triple Incision of miRNA Target: Design Concept and Catalytic Performance

**DOI:** 10.3390/molecules25102459

**Published:** 2020-05-25

**Authors:** Olga Patutina, Daria Chiglintseva, Elena Bichenkova, Svetlana Gaponova, Nadezhda Mironova, Valentin Vlassov, Marina Zenkova

**Affiliations:** 1 Laboratory of Nucleic Acids Biochemistry, Institute of Chemical Biology and Fundamental Medicine SB RAS, Lavrentiev’s ave. 8, 630090 Novosibirsk, Russia; patutina@niboch.nsc.ru (O.P.); dashachiglintseva@gmail.com (D.C.); sveta-mira@yandex.ru (S.G.); mironova@niboch.nsc.ru (N.M.); vvv@niboch.nsc.ru (V.V.); 2 School of Health Sciences, Faculty of Biology, Medicine and Health, University of Manchester, Oxford Rd, Manchester M13 9PT, UK; Elena.V.Bichenkova@manchester.ac.uk

**Keywords:** artificial ribonuclease, oligonucleotide-peptide conjugate, RNA cleavage, oncogenic miRNA, anti-miRNA therapy, RNase H, miRNase

## Abstract

Irreversible destruction of disease-associated regulatory RNA sequences offers exciting opportunities for safe and powerful therapeutic interventions against human pathophysiology. In 2017, for the first time we introduced miRNAses–miRNA-targeted conjugates of a catalytic peptide and oligonucleotide capable of cleaving an miRNA target. Herein, we report the development of Dual miRNases against oncogenic miR-21, miR-155, miR-17 and miR-18a, each containing the catalytic peptide placed in-between two short miRNA-targeted oligodeoxyribonucleotide recognition motifs. Substitution of adenines with 2-aminoadenines in the sequence of oligonucleotide “shoulders” of the Dual miRNase significantly enhanced the efficiency of hybridization with the miRNA target. It was shown that sequence-specific cleavage of the target by miRNase proceeded metal-independently at pH optimum 5.5–7.5 with an efficiency varying from 15% to 85%, depending on the miRNA sequence. A distinct advantage of the engineered nucleases is their ability to additionally recruit RNase H and cut miRNA at three different locations. Such cleavage proceeds at the central part by Dual miRNase, and at the 5′- and 3′-regions by RNase H, which significantly increases the efficiency of miRNA degradation. Due to increased activity at lowered pH Dual miRNases could provide an additional advantage in acidic tumor conditions and may be considered as efficient tumor-selective RNA-targeted therapeutic.

## 1. Introduction

Rapid progress in the study of the role of non-coding RNAs in the functioning of genome has broadened the scope of therapeutic targets for a variety of human diseases. It was established that small regulatory RNAs, microRNAs (miRNAs) coordinate the major biological processes in cells and tissues through regulation of mRNA as a part of miRISC. The main components of the miRISC are TNRC6A, TNRC6B or TNRC6A proteins from GW182 family functioning as scaffold and Ago2 that serves as a regulator of miRNA stability and activity and promotes mRNA cleavage [1]. Binding of Ago2 to miRNA divides it into three functional domains: (1) “seed chamber” corresponding to the seed region of miRNA (nucleotides 2–8 at the 5′-end), (2) “central gate” which matches the region from 9th to 13th nt of miRNA, and (3) “supplementary chamber” that comprises 3′-end of the molecule. Depending on the degree of complementarity between mRNA and miRNA the following scenarios of RNA processing might be realized: (1) steric block of mRNA translation, if duplex forms only in the “seed-chamber” of miRNA/Ago2 complex; (2) mRNA degradation by Ago2, if mRNA binds with the “seed chamber” as well as in the “central gate” of miRNA/Ago2 complex; and (3) miRNA degradation, if mRNA interacts with miRNA in the “seed region” and “supplementary chamber” [2]. Since development of pathological states associated with tumorigenesis is accompanied by manifold increase in oncogenic miRNA expression and interruption of corresponding mRNA levels, investigators considered miRNAs as significant biotargets for treatment of malignant diseases. Design of various oligonucleotide-based constructions for miRNA inhibition is the most actively developing area in miRNA-based therapy [3]. While small RNA zippers, miRNA sponges and miRNA masks were shown to have relatively high anti-miRNA potential [4], antisense oligonucleotides (asONs) remain to be the most effective among all inhibitors. It was shown that miRNA-targeted ONs bind miRNA even inside miRISC in vitro and in vivo, that was in particular demonstrated for anti-miR-21 and anti-miR-122 oligonucleotides [5]. Moreover, asONs may also initiate release of miRNA from complex with Ago2, followed by inhibition of miRISC activity and significant decrease in viability of tumor cells [6,7].

A new, promising strategy for the highly specific inhibition of pathologically overexpressed miRNAs is the use of miRNA-targeted sequence-specific artificial ribonucleases (aRNases) [8,9,10,11,12]. These RNA cleaving compounds are conjugates of an addressing oligonucleotide that selectively binds to an miRNA target and a covalently attached cleaving group that catalyzes the degradation of target RNA molecule. aRNases, as a particular class of future therapeutics, could have tremendous therapeutic potential due to their ability to specifically recognize and quantitively destroy pathogenic RNA sequences. However, given their short length (≤25 nt), miRNA sequences remain particularly challenging for effective suppression by aRNases in a sequence-specific manner. In order to mitigate the small size of miRNA targets, peptide-nucleic acids (PNA) can potentially be used as recognition motifs of such miRNA-targeted aRNases, which were shown to exhibit an increased affinity towards RNA targets [11,12]. Recently, two types of PNA-based aRNases were proposed to target synthetic hsa-miR-1323, known to be overexpressed in neuroblastoma, elongated by 5 nucleotides. Type I represented Cu^2+^-dependent RNase containing HGG peptide as a cleaving group, whereas type II represented a metal-independent RNase containing diethylenetriamine (DETA) as a cleaving domain [12]. These conjugates were shown to engage with miRNA target and perform its effective degradation by either oxidative or acid-base cleavage, with the release of one or two nucleotides from the 3′ elongated end of the synthetic hsa-miR-1323 target. Another example of successful application of the PNA-based sequence-specific aRNases was recently reported [11], when the activity of the metal-independent conjugate of PNA with tris(2-aminobenzimidazole) was investigated against the synthetic analog of miR-20a, the member of the oncogenic cluster miRNA-17~92 and compared back-to-back with the catalytic performance of the corresponding DNA analogue. Both PNA and DNA conjugates were shown to achieve efficient sequence-specific cleavage of RNA target. It was suggested that the cleavage by such conjugates was carried out in RNase A-like manner. Despite the improved affinity of PNA towards target RNA as compared to DNA analogues, the propensity of PNA to form aggregates [11] may significantly reduce their ribonuclease activity and thus pose a major disadvantage. In addition, such ribonucleases do not retain the ability to recruit natural intracellular enzymes, such as RNase H1, which can potentially increase the number of cleavage cycles and thus enhance the rate of target degradation. This represents the major disadvantage of PNA-based aRNases.

Instead, long, continuous stretches of DNA or hairpin sequences could be employed to enable the precise recognition and efficient hybridization with short non-coding miRNA sequences [8,10,12]. We have recently designed miRNA-targeted sequence-specific artificial RNases, termed miRNases, intended to inactivate miR-17 and miR-21 oncogenic targets [8,9]. The miRNase represents a particular class of the peptidyl-oligonucleotide conjugates, consisting of a hairpin-like DNA (to enhance binding affinity) complementary to the 5′-region of miRNA target, and a cleaving peptide containing alternating arginine and leucine residues. The developed miRNases were shown to carry out efficient sequence-selective cleavage of the 3′-end of miRNA targets (bases 15–21), leading to their inhibition and dysfunction in tumor cells [10]. We recently developed an alternative design of “dual” peptidyl-oligonucleotide conjugates that incorporated two oligonucleotide recognition motifs flanking the catalytically active peptide, which was forced to be positioned against the single-stranded RNA region upon hybridization. Ribonuclease potency of conjugates of new structure was demonstrated earlier by targeting tRNA^Phe^ [13]. The remarkably efficient cleavage of the RNA target was achieved for the best structural variant of such Dual conjugates through a mechanistic balance between conformational tuning and base-pair matching [13]. Herein, we applied a similar concept of “dual” conjugation to chemically engineer Dual miRNases against four different miRNA targets, miR-21, miR-17, miRNA-18a and miR-155. In such a design, the catalytic peptide is placed in-between two short miRNA-targeted oligodeoxyribonucleotide recognition motifs, matching the specific miRNA target and containing 2-aminoadenines substitutions to enhance their binding affinity. The advantage of the proposed design is the ability to carry out cleavage of miRNA targets in the central part of their sequences in order to facilitate the dissociation of non-functional fragments. We compare here the specificity and efficiency of Dual miRNases targeted to four oncogenic miRNAs. We also investigate the influence of the nucleotide composition of the central single-stranded gap-region within the miRNA sequences, and the impact of the flanking nucleobases, on the efficiency of the RNA cleavage. The advantages of the “dual” conjugate design for future intracellular application in the presence of RNase H is also assessed and discussed.

## 2. Results

### 2.1. Design Strategy of Dual Conjugates

The success of the “dual” conjugate strategy introduced earlier for sequence-specific recognition and cleavage of RNA sequences, has been previously demonstrated against non-clinical target (i.e., yeast tRNA^Phe^) [13]. Herein, we describe the implementation of such strategy for selective targeting of the pharmaceutically relevant oncogenic miRNAs, miR-21, miR-17, miRNA-18a and miR-155. These miRNAs were chosen as targets for DCs, since they represent multifunctional regulators of cellular processes and their expression is impeded in wide spectrum of pathologies [14,15,16,17,18,19,20] (Table S1).

**Figure 1:**
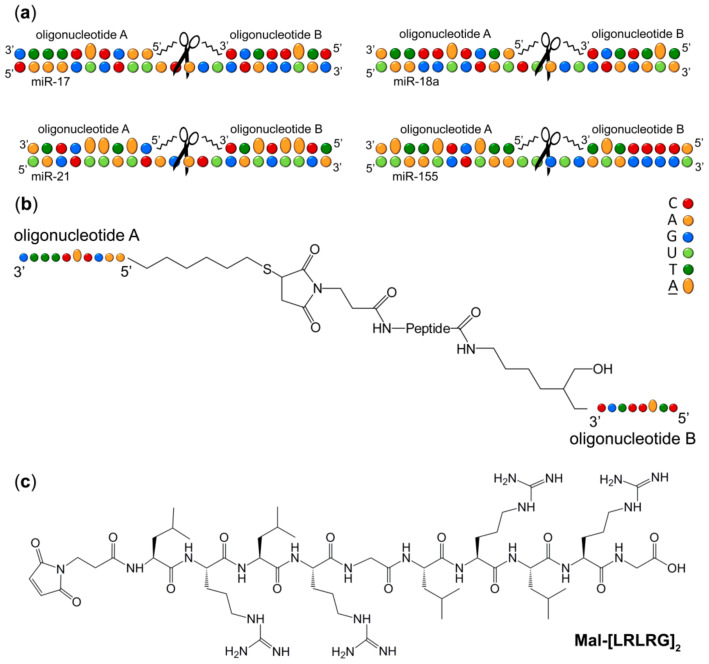
(**a**) Design concept of Dual conjugates, targeted to miR-17, miR-18a, miR-21 and miR-155. A–2-aminoadenine; (**b**) Structure of DC; (**c**) Structure and sequence of the catalytic peptide.

The “dual” conjugate design of miRNA specific peptidyl-oligonucleotide conjugates involves placing of the catalytic peptide [LRLRG]_2_ in-between two short miRNA-targeting deoxyribo-oligonucleotide recognition motifs (Figure 1 and Table 1) to ensure that it is located opposite to the central region of the miRNA sequence. The location of the peptide at the internal position of the conjugates, rather than at the end of the 5′-end of the oligonucleotide recognition motifs [9,10,13,21], provides the opportunity to cleave miRNA approximately in the middle of the chain, leading to a complete loss of normal functioning of the molecule. However, such design requires addressing the key thermodynamic challenges, emerging from targeting relatively short miRNA substrates (≤25 nt), when two recognition motifs of the Dual conjugate need to be short enough (in order to allow 5-nt cleavage site in the middle of miRNA to remain single-stranded), but strong enough in terms of their hybridization ability. To address this challenge, we replaced a few adenines aromatic bases within the conjugate recognition motifs with 2-aminoadenines (Figure 1a, Figure S1 and Table 1) to increase the number of hydrogen bonds, which can be formed between the miRNases and target miRNAs upon hybridization. We anticipated that each substitution of an A-U base pair with a 2-aminoadenine-U base pair could increase the thermostability of the formed DNA:RNA hybrid by approximately 3 °C [22,23]. Moreover, modification with 2-aminoadenines may enhance sequence specificity of the developed compounds. In particular, an introduction of even one mismatched residue in RNA sequence, placed opposite to 2-aminoadenine, was shown to decrease duplex T_m_ from 7 to 15 °C [24]. This suggests an enhancement in a discrimination factor for target RNA, which may restrict binding of 2-aminoadenine-modified compounds to only complementary targets.

Since the catalytic peptide with alternating leucine, arginine and glycine residues [LRLRG]_2_ previously showed efficient cleavage of RNA targets [13,21], we used the same peptide for catalytic destruction of miRNA sequences. Thus, the miRNA-targeted Dual conjugate (here and after DC) consists of two partially modified oligonucleotides (**A** and **B**) complementary to the 5′- and 3′-regions of miRNA target, respectively. The 5′-terminus of the oligonucleotide **A** is connected to the peptide N-terminus via a thiohexyl linker (linker 2), whereas the 3′-terminus of the oligonucleotide **B** is attached to the peptide C-terminus via aminohexyl linker (linker 1). The sequences of the recognition motifs **A** and **B** were selected in such a way that upon hybridization the catalytic peptide becomes adjacent to a 5-nt single-stranded RNA region located in the central part of the target miRNA (Figure 1a). As shown earlier [13], the 5-nt length of a single-stranded RNA region has sufficient flexibility, whereas the adequately long linkers could provide sufficient conformational freedom for the peptide within the hybridized complex for efficient RNA cleavage.

In this study, DCs were designed to target four different therapeutically relevant sequences of highly oncogenic miRNAs, miR-21, miR-155, miR-17 and miR-18a.

The synthesis of DCs was carried out in two subsequent stages [13]. The initial step involved the conjugation of the oligonucleotide **A** to the peptide N-terminus and utilized a thiol-maleimide ‘click’ reaction between 5′-thiol modified oligonucleotide and N-Maleoyl-β-alanine residue (Figure 1) at the peptide N-terminus [25] to generate a Single conjugate (SC). After purification of the SC, the second oligonucleotide **B** bearing a 3′-aminohexyl group was then attached to the peptide C-terminus via an amide coupling reaction to produce the corresponding DC, which was isolated from the reaction mixture by IEX HPLC (Figure S2) and purified further by reversed phase HPLC (Figure S3). The purity of all DCs was assessed by Urea-PAGE (20% PAA/8 M Urea) analysis (Figure S4), and their identity was confirmed by ^1^H-NMR spectroscopy (Figures S5–S8).

### 2.2. Hybridization of Dual Conjugates with miRNA Targets

An indispensable characteristic of aRNase, which has a key effect on the efficiency of sequence-specific cleavage, is its ability to hybridize miRNA target. Our preliminary experiments demonstrated that the separate single (i.e., unconjugated) deoxyribo-oligonucleotides **A** or **B**, which represent the “shoulders” of the corresponding DC (each of 8–10 nt long), were not capable to form complex with their matching miRNA targets at 37 °C (buffer: 50 mM Tris-HCl, pH 7.0, 200 mM KCl and 1 mM EDTA). Dual deoxyribo-oligonucleotides **A** and **B** (ON) connected with a double triethylene glycol linker instead of catalytic peptide, thus imitating the structure of DC, also showed a poor or negligible ability to form complex with miRNA (Figure S9). To overcome this issue and improve the hybridization properties of the conjugates, the adenine aromatic bases located at the internal positions were replaced with the 2-aminoadenines (Figure 1a, Figure S1, and Table 1).

**Figure 2:**
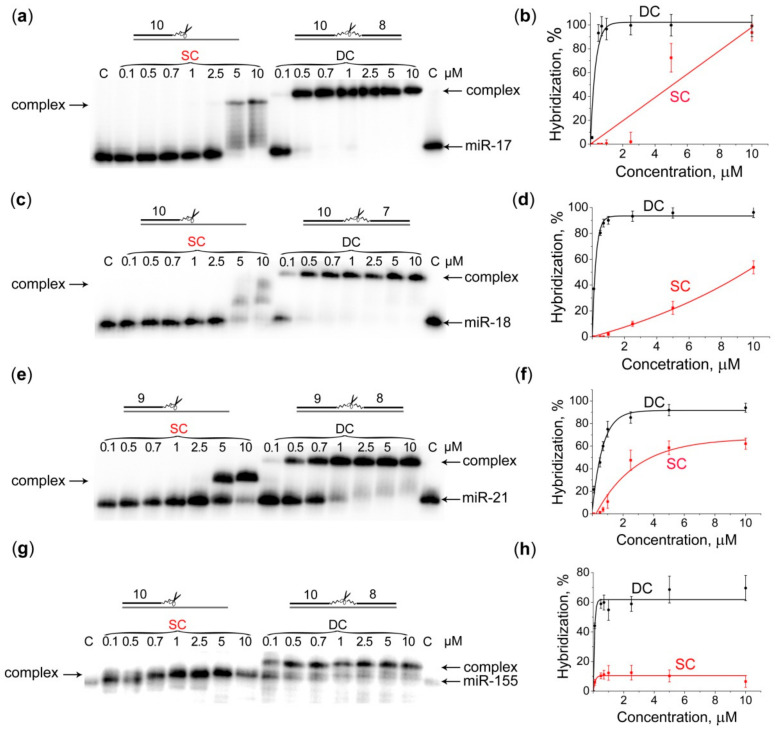
Hybridization of 5′-[^32^P]-labeled miRNAs with Dual (DC) and Single (SC) conjugates. (**a**,**c**,**e**,**g**) Radioautographs of 15% native PAAG, showing hybridization of the corresponding conjugate with miR-17, miR-18a, miR-21 and miR-155, respectively. miRNAs (1 μM) were incubated with conjugates (0.1–10 μM) in buffer (1), containing 50 mM Tris-HCl, pH 7.0, 200 mM KCl and 1 mM EDTA at 37 °C for 45 min. The samples were loaded onto the running gel immediately after quenching the reaction with 1 min intervals. The concentration (μM) of the conjugate is indicated on the top of electrophoregrams; (**b**,**d**,**f**,**h**) Concentration dependencies of SC and DC hybridization with miR-17, miR-18a, miR-21 and miR-155, respectively. Data are presented as mean ± s.e.

We carried out a comparative analysis of the hybridization properties of the developed DCs with the corresponding SCs, which were the products of the first stage of the DCs synthesis. As discussed above, each SC consisted of the corresponding oligonucleotide **A**, which was complementary to the 5′-region of the matching miRNA target, and the catalytic peptide [LRLRG]_2_, attached to the 5′-end of this oligonucleotide via a thiohexyl linker (linker 2). We showed (Figure 2) that all DCs were able to efficiently bind to the matching miRNA targets even at the 1:1 molar ratio. Indeed, the level of binding of DCs to their miRNA targets reached 96, 90, 75, 55% at equimolar concentration (1 μM), and 99, 96, 92 and 69% at 5-fold excess of DCs over miR-17, miR-18a, miR-21 and miR-155, respectively (Figure 2).

In contrast, the corresponding SCs poorly hybridized their miRNA target at the 1:1 molar ratio, and their hybridization efficiency did not exceed 60% even at the 10-fold excess of the conjugate with respect to miRNA (Figure 2). The less efficient binding observed for DC targeted to miR-155 might be the result of spontaneous aggregation of monomeric miR-155 due to the presence of G-rich tetra-nucleotide sequence at the 3′-end of the molecule, which could prevent this target from efficient interaction with the conjugate.

Thus, the data obtained in gel-shift assays demonstrated that DCs were able to recognize their miRNA targets, thus providing the high level of sequence-specificity of interaction.

### 2.3. Cleavage Profile and Kinetics of miRNA Cleavage by Dual Conjugates

**Figure 3:**
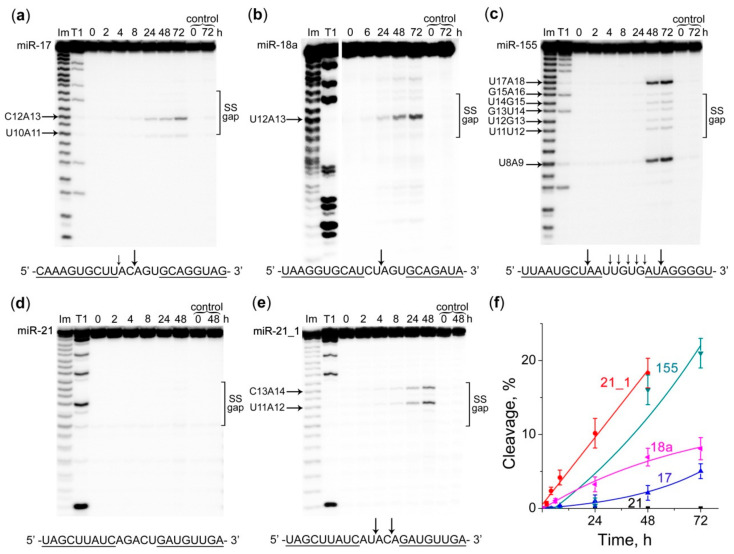
Kinetics of cleavage of 5′-[^32^P]-labeled miR-17, miR-18a, miR-155, miR-21 and miR-21_1 by corresponding DCs in the buffer (1), containing 50 mM Tris-HCl, pH 7.0, 200 mM KCl and 1 mM EDTA. (**a**–**e**) Radioautographs of 18% denaturing PAAG showing the cleavage products of miR-17, miR-18a, miR-155, miR-21 and miR-21_1, respectively. miRNA (1 µM) and corresponding DC (20 µM) were incubated at 37 °C for 72 h. Lanes Im and T1—imidazole ladder and partial RNA digestion with RNase T1, respectively; control–miRNA was incubated in the absence of the conjugate. The incubation time (in hours) is show at the top. SS gap–single-stranded region of miRNA formed upon hybridization with DC; (**f**) Kinetics of miRNAs cleavage by DCs depending on the incubation time. Data are presented as mean ± s.e.

**Figure 4:**
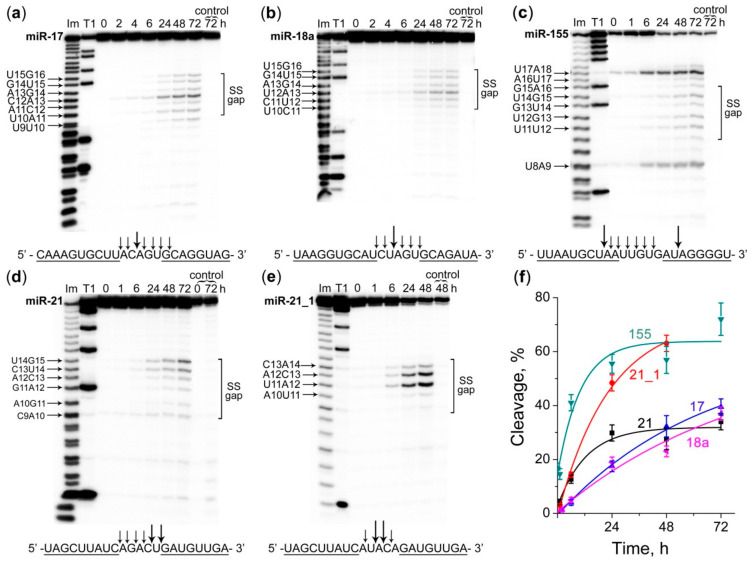
Kinetics of cleavage of 5′-[^32^P]-labeled miR-17, miR-18a, miR-155, miR-21, and miR-21_1 by corresponding DCs in the buffer (2), containing 20 mM Tris-HCl, pH 7.8, 40 mM KCl, 8 mM MgCl_2_, 1 mM DTT, 0.02 mg/mL BSA. (**a**–**e**) Radioautographs of 18% denaturing PAAG showing the cleavage products of miR-17, miR-18a, miR-155, miR-21 and miR-21_1, respectively. miRNA (1 µM) and corresponding DC (20 µM) were incubated at 37 °C for 72 h. Lanes Im and T1–imidazole ladder and partial RNA digestion with RNase T1, respectively; control–miRNA was incubated in the absence of the conjugate. The incubation time (in hours) is shown at the top. SS gap–single-stranded region of miRNA formed upon hybridization with DC; (**f**) Kinetics of miRNAs cleavage by DCs. Data are presented as mean ± s.e.

The ribonuclease activity of DCs was studied under two different buffer conditions: buffer (1) contained 50 mM Tris-HCl, pH 7.0, 200 mM KCl, 1 mM EDTA, which imitates intracellular conditions (e.g., pH and potassium ions concentration) and was used in a number of studies previously [9,10,26,27,28]; and buffer (2) contained 20 mM Tris-HCl, pH 7.8, 40 mM KCl, 8 mM MgCl_2_, 1 mM DTT, 0.02 mg/mL BSA, which is recommended for in vitro reactions with RNase H (Figure 3 and Figure 4). The cleavage of synthetic miRNA targets at a concentration of 1 μM was carried out at a 20-fold excess of the corresponding DCs (20 μM), since in the previous studies these conditions were found to be optimal for comparison of cleaving activity of miRNases [9,10,13].

It was shown that the composition of the buffer could strongly affect the specificity and efficiency of miRNAs cleavage by DCs (Figure 3 and Figure 4). Moreover, ribonuclease assay revealed a clear influence of the sequence of the single-stranded RNA “gap” region formed upon hybridization with the corresponding DC on the cleavage efficiency of a conjugate. It was found that in buffer (1) DCs exhibit predominantly pyrimidine-A cleavage specificity. The main cleavage sites were U10-A11 and C12-A13 for miR-17 (Figure 3a), and U12-A13 for miRNA-18a (Figure 3b). More complex cleavage patterns were observed for miR-155: a weak cleavage was detected for all bonds within the RNA “gap” region, whereas the major cleavage sites included the bonds U8-A9 and U17-A18 within the duplex regions adjacent to the “gap”. Such effect was likely attributed to the duplex thermal “breathing” at the regions flanking the “gap” due to the less stable A:T and U:A base pairs (Figure 3c). Total cleavage extent of miR-17, miRNA-18a and miR-155 in buffer (1) by corresponding DCs is 2%, 7% and 16%, respectively (Figure 3f, Table S2). Unless otherwise specified, here and after the percentages of cleavage efficiency observed after 48 h of reaction are given.

Despite the high binding affinity of all DCs to their matching miRNA targets, miR-21 was not cleaved by the corresponding Dual conjugate (Figure 3d). The most probable reason for this was the absence of the pyrimidine-A sites sensitive towards cleavage by this type of conjugates within the single-stranded “gap” formed upon binding to conjugate. In order to verify this hypothesis, we tested the cleavage of the modified miR-21 sequence (miRNA-21_1) containing 2 substitutions in the gap region (AGACU → AUACA) to generate C-A and U-A bonds. Such replacement, indeed, led to a noticeable cleavage of the miR-21_1 target with miR-21-targeting DC at the introduced U11-A12 and C13-A14 sites (Figure 3e) with the cleavage efficiency reaching 18% (Figure 3f, Table S2).

Remarkably, when cleavage of miRNAs was performed in buffer (2), the cleavage specificity of the studied DCs noticeably changed, and the activity increased significantly. All miRNAs were cleaved at almost every bond within the RNA single-stranded “gap” region with a preference for U-A and C-A sites. The total extent of miRNA cleavage by DCs increased from 2% to 32% for miR-17, from 7% to 23% for miR-18a, and from 16% to 57% for miR-155 (Figure 4a–c, Table S2). Under these conditions the activity of miR-21-targeted DC increased from 0% to 30% for miR-21, and from 18% to 63% for the modified miR-21_1 (Figure 4d,e, Table S2). The observed rate constants (K_obs_) of the RNA cleavage by DCs in buffer (2) raised dramatically by 3-, 6-, 7- and 10-fold for miR-21_1, miRNA-155, miR-18a and miRNA-17, respectively, compared to K_obs_ of cleavage in buffer (1) (Table S2).

Such a strong impact of the buffer composition on the efficiency and patterns of miRNA cleavage prompted us to investigate in detail the key factors (e.g., the concentration of magnesium and potassium ions, buffer pH) influencing the ribonuclease activity of the designed DCs.

### 2.4. The Effect of Mg^2+^ and K^+^ Concentration on the Ribonuclease Activity of Dual Conjugates

It is known that divalent metal ions have a significant effect on the activity of enzymes involved in the nucleic acids metabolism. It has been shown that Mg^2+^ ions can be both activators and inhibitors of intracellular enzymes [26,29,30]. For a number of artificial ribonucleases, the inhibitory effect of Mg^2+^ on the efficiency of substrate cleavage was observed. For non-sequence-specific oligonucleotide-peptide chemical ribonucleases, an increase in magnesium ion concentration from 0 to 5 mM was shown to cause a 2-fold decrease in the degree of substrate cleavage [26]. However, in that case the negative effect of Mg^2+^ on the reaction efficiency of the RNA cleavage by oligonucleotide-peptide conjugates was linked to the increased rigidity of the RNA structure, rather than to the ribonuclease activity of the conjugates.

In order to understand the effect of Mg^2+^ ions on the efficiency and specificity of miRNA cleavage by DCs, miR-18a was incubated with the matching DC in the buffer containing 50 mM Tris-HCl, pH 7.0, 100 mM KCl, 1 mM DTT, 0.02 mg/mL BSA, but with different Mg^2+^ concentration varied from 0 to 15 mM. It was found that, within the experimental error, the addition of Mg^2+^ did not affect the ribonuclease activity of DC which remains approximately 26% (Table S3). These data also indicated that RNA cleavage by DC proceeds metal-independently.

Potassium ions play an important role in the regulation of cell homeostasis by maintaining the membrane potential, cell volume and pH, and may also have an influence on the performance of artificial ribonucleases [31]. Earlier it was demonstrated that oligonucleotide-peptide conjugates exhibit the highest ribonuclease activity at 0–100 mM concentration of K^+^; however, the increase in KCl up to 200 mM lead to significant 3–4-fold drop in RNA degradation efficiency [26]. In order to understand the effect of K^+^ ions on the efficiency of miRNA cleavage by DCs, miR-17 was incubated with the matching DC in the buffer containing 50 mM Tris-HCl, pH 7.0, 1 mM DTT, 0.02 mg/mL BSA and different K^+^ concentration varied from 0 to 400 mM. It was shown that the most potent degradation of miRNA-target, approximately 25%, was observed at 40–100 mM concentration of KCl (Table S4). In the absence of potassium and at a concentration of more than 200 mM, the efficiency of miRNA cleavage was 2-fold reduced.

### 2.5. The Effect of pH on the Ribonuclease Activity of Dual Conjugates

**Figure 5:**
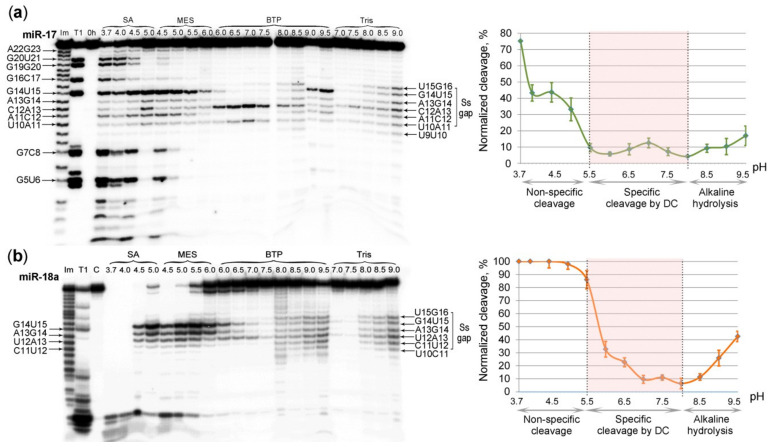
pH profile of 5′-[^32^P]-miR-17 and miR-18a cleavage by DCs. Buffers are listed in the “Materials and methods” section. Radioautograph of 18% denaturing PAAG showing the cleavage products of (**a**) miR-17 and (**b**) miR-18a. miR-17 (1 µM) and miRNA-17-DC (10 µM) or miRNA-18a [1 µM] and miR-18a-DC (20 µM) were incubated at 37 °C for 48 h. Lanes Im and T1–imidazole ladder and partial RNA digestion with RNase T1, respectively; control–miRNA was incubated in the absence of the conjugate in buffer, containing 50 mM Tris-HCl, pH 7.0, 100 mM KCl, 1 mM DTT and 0.02 mg/mL BSA. Cleavage activity of DC at various pH was normalized using values of cleavage activity at overlapping pH values. Pink areas on diagrams show the range of pH at which specific cleavage of miRNAs by DCs was observed. SS gap–single-stranded region of miRNA formed upon hybridization with DC. Data are presented as mean ± s.e.

To investigate the influence of pH on the catalytic performance of the designed DCs, we carried out cleavage of miR-17 and miR-18a by their matching DCs in buffer solutions with the overlapping pH values: sodium acetate (SA) with pH 3.7–5.0; 2-(*N*-morpholino)ethanesulfonic acid (MES) with pH 4.5–6.0; bis-tris-propane-KOH (BTP) with pH 6.0–9.5 and Tris-HCl with pH 7.0–9.0 (for details see “Materials and Methods” section). The cleavage experiments of miR-17 and miR-18a present at a concentration of 1 μM were carried out in different conditions: in a 10-fold excess of miRNA-17-DC (10 μM) and a 20-fold excess of the miR-18a-DC (20 μM). As control experiments, these miRNAs were also incubated in the same buffers under identical conditions, but without the corresponding DCs (Figure S10). The obtained results showed that the activity of DC was weakly dependent upon the nature of the buffer (Figure 5). The scaling factor between the buffers did not exceed 1.4.

Experiments showed that pH profiles of miRNA cleavage by DC could be distinctly divided into three different parts (Figure 5). At the acidic pH (from pH 3.7 to 5.0), the non-specific cleavage throughout the entire sequence of miRNAs was observed (Figure 5a). This might be attributed to the destabilization of miRNA/DC duplex induced by acidic environment. This process was more clearly seen in the case of miR-17, when the molar ratio miRNA:DC was 1:10. In the case of miR-18a, when the molar ratio miRNA:DC was 1:20, miR-18a was almost completely destroyed at these conditions. Interestingly, under these conditions, cleavage occurs predominantly at purine-X sites (G-X and A-X). From pH 3.7 to 6.0, the gradual changes in specificity from purine-X to pyrimidine-X (C-X and U-X) were observed. At the subacid, neutral and subalkaline pH (5.5 to 8.0) the “gap”-selective degradation of miRNAs was detected. The most specific miRNA cleavage by DCs seemed to occur at pH range of 5.5–7.5 for miRNA-17-DC (the most cleavable bonds are U10-A11 and C12-A13) and 6.0–7.5 for miR-18a-DC (the most cleavable bonds are U12-A13, A13-G14 and G14-U15) (Figure 5). Maximum sequence-specific cleavage in the RNA “gap” for miRNA-17-DC and miR-18a-DC was observed at pH values of 7.0 and 5.5, respectively (Figure 5). At the alkaline pH (8.0 to 9.5), a non-specific alkaline hydrolysis of miRNA sequences was observed. At these conditions the cleavage was seen throughout the entire sequence of miRNAs, which was similar to that seen in the control experiments (Figure 5, Figure S10).

### 2.6. Efficiency and Specificity of miRNA Cleavage by Dual Conjugates in the Presence of RNase H

Earlier, we have shown that the efficiency of miRNA degradation by a miRNase increases significantly in the presence of RNase H due to the synergistic nature of their joint action [8,9]. It is known that DNA-like antisense oligonucleotides after binding to target RNAs form a substrate for intracellular endonuclease RNases H. RNA hydrolysis in RNA-DNA heteroduplex by RNase H1 occurs at a distance of 6–10 nucleotides from the 5′-end of RNA (approximately one turn of the helix) [32]. Our design of the Dual conjugates here was carried out in such a way to ensure that their heteroduplexes with the matching miRNA sequences could serve as potential substrates for RNase H. To achieve that, we ensured that the length of the oligonucleotide recognition motifs **A** and **B** within the DC was 8–10 nt, which was expected to be sufficient for interaction and cleavage by RNase H (see Figure 1 and Table 1). As indicated above, a few adenine aromatic basis within each recognition motifs **A** and **B** were replaced with 2-aminoadenines to enhance the hybridization ability of the DC with miRNA. It was reported earlier that incorporation of 2-aminoadenines into antisense oligonucleotide did not interference with RNase H activity [33]. In order to verify this, as well as to evaluate the ability of RNase H to contribute to the miRNA cleavage catalyzed by DC, we studied the kinetics of the target miRNA cleavage performed (1) by the DC alone; (2) by RNase H, when the target miRNA was hybridized with the nonconjugated Dual oligonucleotide (ON); and (3) by both DC and RNase H present in the same reaction mixture. The study was performed against miR-17, miR-155 and miR-21 using a 20-fold excess of DCs or respective oligonucleotides over the matching miRNA (see Table 1 for sequences).

**Figure 6:**
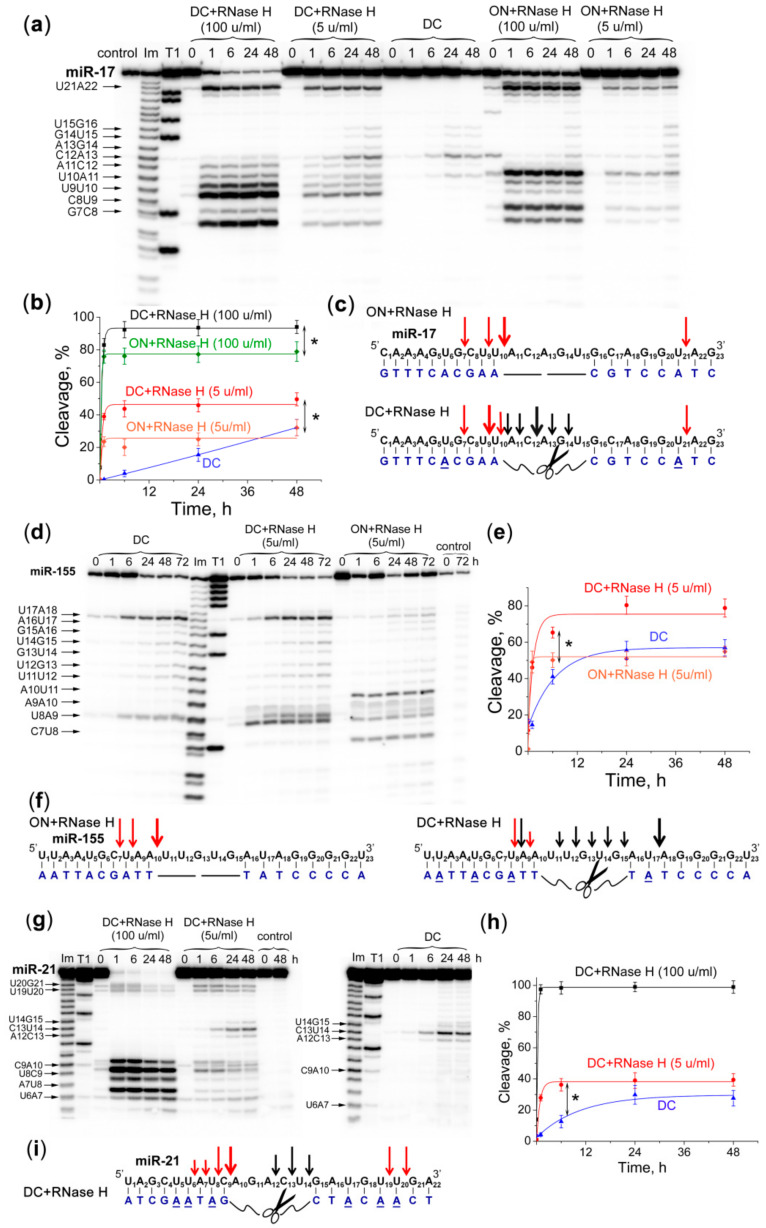
Cleavage kinetics of 5′-[^32^P]-labeled miRNAs by DCs and/or RNase H. (**a**,**d**,**g**). Radioautographs of 18% denaturing PAAG showing the profiles of cleavage of miR-17, miR-155 and miR-21, respectively. Duplexes formed by 5′-[^32^P]-miRNA (1 µM) and DC or ON (20 µM) were incubated at 37 °C for 48–72 h. RNase H was taken in the concentration of 5 or 100 u/mL. Lanes Im and T1–imidazole ladder and partial RNA digestion with RNase T1, respectively; control–RNA was incubated in the absence of DC or ON and in the presence of RNase H (100 u/mL); (**b**,**e**,**h**) Time dependency of cleavage of miR-17, miR-155 and miR-21, respectively, by DCs and/or RNase H. Data are presented as mean ± s.e. *—indicates statistically significant difference with *p* < 0.05; (**c**,**f**,**i**). Positions of miR-17, miR-155 and miR-21 cleavage, respectively, induced by RNase H in duplex with ON (red arrows), and by combination of DC (black arrows) and RNase H. A–2-aminoadenine.

Cleavage studies confirmed that 2-aminoadenines did not interfere with the recognition and cleavage of the heteroduplexes miRNA/DC by RNase H. The cleavage of miRNAs by RNase H was observed at the nucleotide positions of 6–10 and 19–21; that is, at the bases 6–10 from the 3′-end of each oligonucleotide “shoulder” (Figure 6).

The study of miR-17 cleavage by RNase H showed that the patterns of target cleavage in the heteroduplexes with ON and DC differ (Figure 6a,c). In the duplex with ON, RNase H cleaves miR-17 predominantly at G7-C8, U10-A11 and U21-A22 sites. Upon incubation of miRNA with DC and RNase H, in addition to cleavage sites attributed to the action of RNase H, the new cleavage sites appeared, which were specific for cleavage by DC, namely, U10-A11, A11-C12 and C12-A13 (Figure 6a,c). Kinetics of miR-17 cleavage showed that at both concentrations of RNase H (i.e., 5 and 100 u/mL) the total efficiency of miR-17 cleavage in the complex with DC was more than 1.5-fold higher than that seen for the miRNA/ON complex. Kinetics of miRNA cleavage in the presence of RNase H reaches a plateau less than in 2 h, and by this time the total extent of miRNA cleavage by the mixture of DC and RNase H (at 5 and 100 u/mL concentration) was 44 and 92%, respectively (Figure 6b), while the total cleavage of the miRNA/ON complex by RNase H was only 25 and 76%, respectively (Figure 6b). It should be emphasized that simultaneous action of miRNA-17-DC and RNase H promotes tremendous increase in the rate of miR-17 cleavage by up to 10-fold after 1 h as compared with the catalytic action of DC alone, and by 1.5-fold as compared with the individual cleavage of the same target by RNase H alone.

The major sites of miR-155 cleavage in the heteroduplex with ON by RNase H are U17-A18, A10-U11, U8-A9 and C7-U8 (Figure 6d,f). Upon incubation of miR-155 with both DC and RNase H, the additional cleavage sites appeared, which are characteristic for the catalytic action of miRNA-155-DC, namely, U17-A18, U8-A9 and A9-A10, with some variations in the extents of cleavage as compared with the individual performance of DC and RNase H against miR-155 (Figure 6d,f). Similar to the cleavage of miR-17 carried out by RNase H at 5 u/mL concentration, the total extent of miR-155 cleavage in the complex with DC was almost 1.5-fold higher than that seen for the miRNA/ON complex. Simultaneous cleavage of miR-155 with DC and RNase H allowed to reach a plateau within 6 h, and the maximum extent of miR-155 cleavage (75%) was about 2-fold higher than that of the individual action of DC against the same target (Figure 6e).

The combined action of DC and RNase H was also studied against miR-21 target (Figure 6i). When the concentration of RNase H was low (i.e., 5 u/mL), the total extent of miR-21 cleavage by DC and RNase H was only 40% (Figure 6i). However, at these conditions, we could clearly observe that the cleavage products of miR-21 fall into three categories: (1) the products from cleavage of miRNA by DC within a single-stranded “gap” region (i.e., sites A12-C13, C13-U14 and U14-G15); (2) and (3) the products resulted from the cleavage of miR-21 by RNase H at the 5′- and 3′-regions of miRNA in heteroduplexes with the recognition motifs **A** and **B** (i.e., sites U6-A7, U8-C9, C9-A10, U19-G20 and U20-G21) (Figure 6h,g). The rate of miR-21 cleavage by miRNA-21-DC increases 4-fold in the presence of RNase H (5 u/mL). With an increase in RNase H concentration to 100 u/mL, a 100% cleavage of miR-21 was dominated by this enzyme and completed in the first hour of incubation (Figure 6h,i). The rate of miR-21 cleavage increases 20-fold compared with miRNA-21-DC alone.

## 3. Discussion

Over recent years, interest in the development of oligonucleotide derivatives for the suppression of hyperexpressed pathogenic RNAs has grown significantly. In the last three years several novel types of oligonucleotide conjugates for targeted degradation of tumor-associated RNA targets were designed, which included Zn^2+^- and Cu^2+^-dependent conjugates of the short peptide nucleic acids (PNA) and neocuproine for degradation of Leukemia-related bcr/abl mRNA fragment [34,35]; metal-independent conjugates of DNA/LNA mixmers with tris(2-aminobenzimidazole), capable of cleaving the proto-oncogenic serine-threonine kinase PIM1 mRNA fragment [36]; and conjugates of hairpin DNA or 2′-OMe oligonucleotides and a peptide [LRLRG]_2_, so-called miRNAses, for selective inactivation of oncogenic miRNAs miR-17 and miR-21 [8,9,10].

We recently proposed an innovative way of incorporating the RNA-cleaving moiety into the structure of the peptidyl-oligonucleotide conjugates, when the catalytic peptide [LRLRG]_2_ was placed in-between two separate recognition motifs to form a Dual conjugate [13]. In the present study this “dual” conjugation strategy against of therapeutically relevant oncogenic miRNAs, miR-21, miR-17, miR-18a and miR-155 was applied. We designed Dual miRNases for selective destruction of their matching miRNA targets in the central part of the sequence. Investigation of miRNA cleavage by these four conjugates was carried out in two buffer systems contained 50 mM Tris-HCl, pH 7.0, 200 mM KCl, 1 mM EDTA, buffer (1); or 20 mM Tris-HCl, pH 7.8, 40 mM KCl, 8 mM MgCl_2_, 1 mM DTT, 0.02 mg/mL BSA, buffer (2). It was found that in buffer (1) DCs exhibit weak ribonuclease activity, whereas in buffer (2) efficiency of miRNA degradation is superior. Comparison of buffer solutions revealed the components and factors that potentially may have impact on activity of conjugates, in particular: (1) Mg^2+^and K^+^; (3) pH of media, (4) dithiothreitol (DTT), and (5) bovine serum albumin (BSA).

About 90% of magnesium in an eukaryotic cell exists in the bound form (in a form of complexes with nucleic acids, ATP, negatively charged phospholipids and proteins), and only 10% of Mg^2+^ are in a free form [37]. The intracellular concentration of free magnesium is 0.5–1 mM. No influence of Mg^2+^ at the concentrations ranging from 0 to 15 mM on DC cleavage activity was observed, even at highest concentration representing extreme 15-fold excess of the cations compared to cell content. Thus, it can be assumed that in the intracellular condition, DCs will work independently from magnesium cations.

The optimal concentration of K^+^ for DC activity was found to be 100 mM, which concurs with an average intracellular amount of potassium in mammalian cells (100–140 mM) [38,39]. These data suggest that cellular environment will provide favorable conditions for the catalytic performance of DCs.

The study of DCs activity in various buffers with different pH revealed considerable differences both in the patterns and efficiency of target miRNA degradation. In acidic conditions (pH 3.7–5.0) the non-specific hydrolysis of bonds within the miRNA sequence was observed. According to the published data, decrease in pH may result in the drop of duplex melting temperature (Tm). As it was seen for 13-mer RNA duplexes alteration of pH from 6.1 to 3.6 led to the reduction of Tm from 65.7 to 35.5 °C, with RNA strands being protonated and separated [40]. This suggested that at pH 3.7–5.0, Tm of miRNA/DC heteroduplexes seemed to decrease, thus leading to the complex destabilization. As the result, the entire miRNA sequence became cleaved by the conjugate peptide. In acidic conditions (pH 4–5) phosphodiester bonds are the most stable; therefore, self-cleavage of single-stranded RNA at this pH was unlikely and excluded using an independent control experiment (Figure S10) [41]. At the neutral pH region (5.5 to 8.0) the maximal sequence-specific cleavage of miRNA by DC in the single-stranded “gap” formed upon duplex formation is observed, since the miRNA/DC complex at this pH is the most stable. In the alkaline conditions (pH more than 8.0) cleavage of miRNA became non-specific again: duplex stability is reduced because of the deprotonation of DNA molecule and dissociation of oligonucleotide component of conjugate from RNA target [42]. As a result RNA, possessing the intramolecular nucleophile–2′-OH group, undergoes the self-cleavage in alkaline conditions (Figure S10) [43]. It should be noted that in the pH range studied (3.7–9.5) the charge of the peptide in the DC does not influence the efficiency of miRNA cleavage, since guanidine group of arginine remained equally protonated at all pH values (pKa of arginine amounts to 13.8 ± 0.1) [44]. Thus, the required sequence-specific degradation of miRNA molecule is observed at subacidic and neutral pH.

It was reported that pH of extracellular tumor environment, especially, in hypoxic and anoxic tumor tissue areas, is subacidic and varies from 5.4 to 6.8 depending on the type of human neoplasia, compared with pH 7.0–7.6 in normal neighboring tissues [45,46]. It is mostly contributed to high production of lactic acid and intensive function of Na^+^/K^+^-ATPases and proton pump [47]. The data on pH profiling clearly indicates that the designed DCs will attack miRNAs preferentially in acidic tumor environment and with less efficiency in surrounding normal tissues. Unlike most chemotherapy drugs, the therapeutic effect of which is significantly reduced or eliminated under acidic condition of neoplasia [48], the efficiency of artificial Dual miRNases does not only decrease, but will be significantly potentiated, leading to potent retardation of tumor growth and inhibition of the invasiveness and metastatic capacity of tumor cells. Due to the increased activity at lowered pH, Dual conjugates can be tumor-selective and effective even at early stages of tumor disease, since acidic hypoxic and anoxic tumor regions are already present at early-growth stages and expanded with tumor growth [46].

DTT represents a reducing agent that prevents formation of intramolecular S-S bridges in the active conformation of enzymes [49]. The impact of DTT on cleavage efficiency of DCs was not detected, that could be explained by the lack of the cysteine residues in the structure of cleaving peptide of DCs.

Another buffer component that may affect the ribonuclease activity of DC is BSA. According to literature data, BSA is able to stabilize and enhance the activity of several natural enzymes including cellulose and restriction endonucleases, as well as commercially used proteases and lipases within cross-linked enzyme aggregates (CLEA) [50,51,52]. BSA possesses a high amount of positively charged amino acids lysines in its structure which defines the possible mechanisms of enzyme stabilization [51]. In particular, BSA may prevent the thermal denaturation of enzymes through direct binding and maintenance of their active conformation [52]. Serum albumins are also known to bind with nucleic acids via electrostatic interactions [53]. In case of Dual conjugates, BSA most likely enhances their performance by direct binding with oligonucleotide components of DCs thereby preventing the formation of intermolecular aggregates between negatively charged nucleic acid and positively charged catalytic peptide of DCs. Indeed, addition of BSA to the reaction provides 1.5-fold increase in the cleavage efficiency of DC (Figure S8). Moreover, under in vivo conditions binding of Dual conjugates to serum albumin can significantly increase the nuclease resistance of compounds, prolonging their therapeutic effect [54].

An interesting characteristic of Dual miRNases is their base-specificity. In previous studies, we showed that the most active tRNA-targeted Dual conjugate is characterized by a moderate cleavage rate at pyrimidine-X bonds in the single-stranded gap and a high cleavage rate at G-X bonds in remote areas outside the target region [13]. When targeting to short RNA targets, moderate cleavage of miRNAs mainly at pyrimidine-X bonds in the single-stranded gap is observed (at neutral pH conditions). Taking into account the data obtained, it can be assumed that such Dual conjugates can be efficient against long structured molecules, such as clinically significant mRNAs, long non-coding RNAs and pre-miRNA, due to their ability for multiple cleavage of target molecules. The hairpin structure of long non-coding RNAs and pre-miRNAs makes interactions with Dual conjugates challenging, thus requiring an additional step of structure unfolding prior to binding and cleavage. The solution to this problem could be the use of longer oligonucleotide recognition shoulders in Dual conjugate design to ensure that they are capable of unfolding RNA internal structures. However, additional investigations in this direction are required.

The amphipathic nature of the peptide component within the conjugates, and the fact that each conjugate contains both positively-charged catalytic peptide and negatively-charged oligonucleotides, may suggest that such structures may potentially form inter-molecular assemblies. Indeed, we have recently shown by NMR Diffusion Ordered Spectroscopy [55] that in de-ionized water (e.g., in the absence of counter ions) the short “single” peptidyl-oligonucleotide conjugates may self-assemble into dimers, presumably via electrostatic interactions between the positively-charged peptide of one molecule and the negatively-charged oligonucleotide of a neighboring molecule. However, in contrast to the aggregation downsides reported for PNA conjugates, such self-assembly of the peptidyl-oligonucleotide conjugates into supra-molecular structures might be beneficial as a key factor contributing to catalysis. Indeed, there were several reports of the non-linear enhancement of the catalytic activity of such conjugates with concentration [8,10,13,56] when possible formation of catalytic multiplexes was suggested to play an important factor in the catalytic transesterification of RNA sequences.

Among the properties providing long-lasting and stable effect of therapeutic agents in cells, resistance to nuclease degradation represents a vital characteristic of the developed compounds. Nuclease resistance studies conducted for miRNA-155-DC and miRNA-21-DC showed that in 10% fetal bovine serum (FBS) the developed DCs demonstrated relatively high stability: the half-life times (τ_1/2_) of miRNA-21-DC and miRNA-155-DC were found to be 8 ± 0.1 h and 9.01 ± 0.5 h, respectively (Figure S12). These values considerably surpassed τ_1/2_ of the corresponding oligonucleotides that were found to be 0.75 ± 0.09 and 0.5 ± 0.06 for miRNA-21-ON and miRNA-155-ON, respectively (Figure S12). Moreover, the stability of designed DCs is comparable with the nuclease resistance of the 2’OMe oligonucleotides under the same conditions [57]. Thus, the presence of the 2-aminoadenines and the catalytic peptide in the structure of the DCs seemed to provide sufficient stability against cellular nucleases, thus suggesting the possibility of long-term miRNA downregulation in tumor cells.

Particularly noteworthy are experiments using the combination of DC and RNase H–conditions that mimic the intracellular environment. It is known that the main types of modifications of the nucleotide bases in the structure of oligonucleotides, such as O-Methoxyethyl (MOE), locked nucleic acid (LNA) or constrained ethyl (cEt), which stabilize duplex formation with the target RNA, are not compatible with RNase H functioning [58]. A replacement of several adenosine aromatic bases in the conjugate recognition motifs with 2-aminoadenines allowed us not only to achieve highly efficient binding to the target miRNA sequences, but also to preserve RNase H-engaging ability, which significantly increased the rate of miRNA degradation. Experiments have shown that the presence of a peptide group in the conjugate changes the main pattern of RNase H cleavage, since a shift of the region accessible for RNase H towards the 5′-end of miRNA was observed: in the complexes miR-17/ON and miR-155/ON, the main bond was U10-A11, and in the complexes miR-17/DC and miR-155/DC the main cleavable bond was U9-U10 and U8-A9, respectively. We may assume that further DC and RNase H function independently. In the first hours of the reaction, when the main miRNA cleavage was observed, the effect of nucleases could be summed up. The observed additive effect of the natural and artificial enzymes seemed to be implemented by cutting the miRNA molecule at three independent regions, i.e., in the 5′- and 3′-regions by RNase H, and in the central part by DC. Such degradation of the target miRNA certainly leads to a complete loss of the regulatory activity of the molecule, since it affects the key nucleotide motifs of miRNAs necessary for effective repression of target mRNA: canonical 7-mer seed site (2–7 nucleotides) and “3′-compensatory” or “beneficial 3′-paring” site (nucleotides 12–17) responsible for mRNA target recognition and downregulation [59,60,61,62]. As a result of the combined action of DC and RNase H, the miRNA region from nucleotide 6–7 to 14–17 appears to be completely destroyed.

In comparison to the previously developed miRNases, consisting of a hairpin oligonucleotide and a peptide attached at the 5′-end of the guiding sequence and initiating miRNA degradation from its 3′-region [8,10], the Dual conjugates are designed to cut the miRNA in its central part, which is expected to lead to a complete loss of the key functionality of this regulatory RNA, thus providing a greater therapeutic potential. We expect that the irreversible damage of miRNA in the region corresponding to “central gate” of miRNA/Ago2 complex (the 9–13 nt site of miRNA sequences) will strongly affect the biological performance of the oncogenic miRNA molecules and may lead to a complete knockdown. Moreover, inside the cell the triple incision of miRNA sequence, achieved here with the additional recruitment of intracellular RNase H, offers the opportunity for a triple attack on the target miRNA sequences. This may trigger a simultaneous destruction of the three key functional regions within the target miRNA, matching to “seed chamber”, “central gate” and “supplementary chamber”, thus leading to a complete demolition of pathogenic miRNAs. This may also facilitate the dissociation and release of cleaved miRNA fragments from the complex with the conjugate, leading to a next “binding-cleavage” cycle to enhance further the therapeutic potency.

Previously, we showed that the hairpin miRNases were able to perform specific deactivation of miR-21 in tumor cells, which triggered a broad spectrum of biological responses, thus leading to the inhibition of pro-survival behavior of tumor cells, including induction of apoptosis and suppression of cell invasiveness. Furthermore, it was shown that treatment of tumor cells with miR-21-miRNase prior to transplantation into mice, led to almost complete cessation of tumor growth [8]. In parallel with this, miRNases did not cause any detectable non-specific toxic effects in cells. These suggested that Dual miRNases representing the same class of miRNA-targeted artificial ribonucleases, may have high therapeutic potential in vivo, provided that the requirements of increased stability, good bioavailability and low toxicity are met.

## 4. Materials and Methods

### 4.1. Oligonucleotides

Dual oligodeoxyribonucleotides with double triethylene glycol linker were synthesized in the Laboratory of Medicinal Chemistry, ICBFM, Russia, by the standard phosphoramidite protocol [63,64,65] on an ASM-800 synthesizer (Biosset, Novosibirsk, Russia) using solid support, nucleoside phosphoramidites and chemical phosphorylation reagent from Glenn Research (Sterling, VA, USA). Oligonucleotides were isolated by consecutive ion-exchange (Polysil SA-500 columns, “Vector”, Koltsovo Village, Russia) and reverse-phase HPLC (LiChrosorb RP-18 columns, Merck, Kenilworth, NJ, USA) according to standard protocols. 5′-thiohexyl and 3′-aminohexyl oligonucleotides containing 2-amino-adenosines were purchased from ATDBio Ltd., Southampton, UK. The sequences of oligonucleotides are given in Table 1.

### 4.2. Synthesis of the Conjugates

The peptide Mal-[LRLRG]_2_ was purchased from Almabion, Voronezh, Russia. The synthesis of each Dual conjugate (DC) was carried out using two-step procedure. First, the synthesis of the corresponding Single conjugate was completed using 5′-thiohexyl oligonucleotides and maleimide-modified peptide, Mal-[LRLRG]_2_ via a thiol-maleimide ‘click’ reaction. This was followed by the synthesis of the corresponded Dual conjugate employing the isolated and purified Single conjugate generated in the first step and the corresponding 3′-aminohexyl oligonucleotide. Synthetic strategy and purification conditions of DC were described in details in [13].

Purifications of the conjugates was carried out using Agilent 1100 HPLC system (Agilent Technologies, Santa Clara, CA, USA), with the UV absorbance being monitored at 260 nm. Ion-exchange (IEX) HPLC purification was employed both to isolate the Single conjugates generated during the first step of the synthesis, and to separate the Dual conjugates from the precursors after the second step (see Figure S2). IEX was performed in a similar way as described earlier [13] using a Clarity Oligo-WAX (150 × 4.6 mm, 10 Å pore size, Phenomenex; Torrance, CA, USA) column. This was followed by reversed phase (RP) HPLC purification of the Single and Dual conjugates (see Figure S3) using a semi-preparative column Luna C18 (Phenomenex; Torrance, CA, USA) and a previously described purification protocol [21]. The excess salt was removed by gel filtration chromatography using IllustraTM NAP disposable columns prepacked with Sephadex G-25 (DNA grade resin) to remove any possible traces of counter cations from peptidyl-oligonucleotide conjugates. The homogeneity and purity of the Dual conjugates miRNA-155-DC, miRNA-18a-DC, miRNA-21-DC and miRNA-17-DC were tested by Urea-PAGE (20% PAA/8 M Urea) analysis (see Figure S4), and the purity of the conjugates was confirmed to be between 95% and 97%. Urea-PAGE analysis also demonstrated the reduced electrophoretic mobility of the Dual conjugates as compared to that for the corresponding Single conjugates miRNA-155-SC, miRNA-18a-SC, miRNA-21-SC and miRNA-17-SC, respectively (Figure S4). The identity of all DCs has been confirmed by ^1^H-NMR spectroscopy (Figures S5–S8).

^1^H spectra were recorded using Bruker Avance II+ 400 NMR spectrometer (400 MHz) equipped with TopSpin 3.2 software for NMR data acquisition and processing (Bruker, Billerica, MA, USA). However, the H3’/H4’/H5’/H5” sugar ring proton regions (3.5–5.0 ppm) were not analysed due to suppression of residual water signal at 4.8 ppm.

**miRNA-21-SC:**^1^H-NMR (D_2_O with TSP (0.01 μM), 400 MHz): δ 0.70–0.99 (m, 24H, Leu-H^δ^), 1.25–3.25 (m, 74H, 9 × H2’ and 9 × H2” sugar ring protons, 6H of 2 × dT (CH_3_), 8 × Arg-H^β^, 8 × Arg-H^γ^, 8 × Leu-H^β^, 4 × Leu-H^γ^, 8 × Arg-H^δ^, 12H of 6 × CH_2_ (thiohexyl linker) and 2 × Mal-H^β^), 5.40–6.33 (m, 10H, 9 × H1’ sugar ring protons, 1 × H5 of dC), 7.25–8.41 (m, 10H, 10 × Ar-H from dG (×2), dA (×2), 2-amino-dA (×3), dC (×1) and dT (×2)).

**miRNA-21-DC:**^1^H-NMR (D_2_O with TSP (0.01 μM), 400 MHz): δ 0.20–0.65 (m, 7H, 6H of 3 × CH_2_ (aminohexyl linker) and 1 × CH (aminohexyl linker), 0.70–0.99 (m, 24H, Leu-H^δ^), 1.25–3.25 (m, 102 H, 17 × H2’ and 17 × H2” sugar ring protons, of 12H of 4 × dT (CH_3_), 8 × Arg-H^β^, 8 × Arg-H^γ^, 8 × Leu-H^β^, 4 × Leu-H^γ^, 8 × Arg-H^δ^, 6H of 3 × CH_2_ (aminohexyl linker), 12H of 6 × CH_2_ (thiohexyl linker) 2 × Mal-H^β^), 5.40–6.33 (m, 21H, 17 × H1’ sugar ring protons, 4 × H5 of dC), 7.25–8.41 (m, 18H, 18 × Ar-H from dG (×2), dA (×2), 2-amino-dA (×6), dC (×4) and dT (×4)).

**miRNA-17-SC:**^1^H-NMR (D_2_O with TSP (0.01 μM), 400 MHz): δ 0.70–0.99 (m, 24H, Leu-H^δ^), 1.25–3.25 (m, 79H, 10 × H2’ and 10 × H2” sugar ring protons, 9H of 3 × dT (CH_3_), 8 × Arg-H^β^, 8 × Arg-H^γ^, 8 × Leu-H^β^, 4 × Leu-H^γ^, 8 × Arg-H^δ^, 12H of 6 × CH_2_ (thiohexyl linker) and 2 × Mal-H^β^), 5.40–6.33 (m, 12H, 10 × H1’ sugar ring protons, 2 × H5 of dC), 7.25–8.41 (m, 11H, 11 × Ar-H from dG (×2), dA (×2), 2-amino-dA (×2), dC (×2) and dT (×3)).

**miRNA-17-DC:**^1^H-NMR (D_2_O with TSP (0.01 μM), 400 MHz): δ 0.70–0.99 (m, 31H, 24H of 4 × Leu-H^δ^, 6H of 3 × CH_2_ (aminohexyl linker) and 1 × CH (aminohexyl linker)), 1.25–3.25 (m, 107 H, 18 × H2’ and 18 × H2” sugar ring protons, of 15H of 5 × dT (CH_3_), 8 × Arg-H^β^, 8 × Arg-H^γ^, 8 × Leu-H^β^, 4 × Leu-H^γ^, 8 × Arg-H^δ^, 6H of 3 × CH_2_ (aminohexyl linker), 12H of 6 × CH_2_ (thiohexyl linker) 2 × Mal-H^β^), 5.40–6.33 (m, 24H, 18 × H1’ sugar ring protons, 6 × H5 of dC), 7.25–8.41 (m, 19H, 19 × Ar-H from dG (×3), dA (×2), 2-amino-dA (×3), dC (×6) and dT (×5)).

**miRNA-18a-SC:**^1^H-NMR (D_2_O with TSP (0.01 μM), 400 MHz): δ 0.70–0.99 (m, 24H, Leu-H^δ^), 1.25–3.25 (m, 79H, 10 × H2’ and 10 × H2” sugar ring protons, 9H of 3 × dT (CH_3_), 8 × Arg-H^β^, 8 × Arg-H^γ^, 8 × Leu-H^β^, 4 × Leu-H^γ^, 8 × Arg-H^δ^, 12H of 6 × CH_2_ (thiohexyl linker) and 2 × Mal-H^β^), 5.40–6.33 (m, 13H, 10 × H1’ sugar ring protons, 3 × H5 of dC), 7.25–8.41 (m, 12H, 12 × Ar-H from dG (×1), dA (×4), 2-amino-dA (×1), dC (×3) and dT (×3)).

**miRNA-18a-DC:**^1^H-NMR (D_2_O with TSP (0.01 μM), 400 MHz): δ 0.70–0.99 (m, 31H, 24H of 4 × Leu-H^δ^, 6H of 3 × CH_2_ (aminohexyl linker) and 1 × CH (aminohexyl linker)), 1.25–3.25 (m, 108 H, 17 × H2’ and 17 × H2” sugar ring protons, of 18H of 6 × dT (CH_3_), 8 × Arg-H^β^, 8 × Arg-H^γ^, 8 × Leu-H^β^, 4 × Leu-H^γ^, 8 × Arg-H^δ^, 6H of 3 × CH_2_ (aminohexyl linker), 12H of 6 × CH_2_ (thiohexyl linker) 2 × Mal-H^β^), 5.40–6.33 (m, 22H, 17 × H1’ sugar ring protons, 5 × H5 of dC), 7.25–8.41 (m, 19H, 19 × Ar-H from dG (×2), dA (×4), 2-amino-dA (×2), dC (×5) and dT (×6)).

**miRNA-155-SC:**^1^H-NMR (D_2_O with TSP (0.01 μM), 400 MHz): δ 0.70–0.99 (m, 24H, Leu-H^δ^), 1.25–3.25 (m, 82H, 10 × H2’ and 10 × H2” sugar ring protons, 12H of 4 × dT (CH_3_), 8 × Arg-H^β^, 8 × Arg-H^γ^, 8 × Leu-H^β^, 4 × Leu-H^γ^, 8 × Arg-H^δ^, 12H of 6 × CH_2_ (thiohexyl linker) and 2 × Mal-H^β^), 5.40–6.33 (m, 11H, 10 × H1’ sugar ring protons, 1 × H5 of dC), 7.25–8.41 (m, 11H, 11 × Ar-H from dG (×1), dA (×2), 2-amino-dA (×3), dC (×1) and dT (×4)).

**miRNA-155-DC:**^1^H-NMR (D_2_O with TSP (0.01 μM), 400 MHz): δ 0.70–0.99 (m, 31H, 24H of 4 × Leu-H^δ^, 6H of 3 × CH_2_ (aminohexyl linker) and 1 × CH (aminohexyl linker)), 1.25–3.25 (m, 110 H, 18 × H2’ and 18 × H2” sugar ring protons, of 18H of 6 × dT (CH_3_), 8 × Arg-H^β^, 8 × Arg-H^γ^, 8 × Leu-H^β^, 4 × Leu-H^γ^, 8 × Arg-H^δ^, 6H of 3 × CH_2_ (aminohexyl linker), 8H of 4 × CH_2_ (thiohexyl linker) 2 × Mal-H^β^), 5.40–6.33 (m, 23H, 18 × H1’ sugar ring protons, 5 × H5 of dC), 7.25–8.41 (m, 20H, 20 × Ar-H from dG (×1), dA (×4), 2-amino-dA (×4), dC (×5) and dT (×6)).

### 4.3. RNA Labelling

5′-end labelling using [^32^P]-ATP and T4 polynucleotide kinase (Thermo Scientific, Waltham, MA, USA) and isolation of [^32^P]-miRNAs miR-21, miR-21_1, miR-17, miR-18a, miR-155 (Table S5) were carried out according to procedure previously described in [26,66].

### 4.4. Gel-Retardation assay

The reaction mixture (4 µL) containing 50 mM Tris-HCl, pH 7.0, 200 mM KCl and 1 mM EDTA, 100 cpm (Cherenkov’s counting) of [^32^P]-miRNA, 1 μM unlabeled miRNA, a conjugate at a concentration ranging from 0.1 to 10 μM was incubated at 37 °C for 45 min and quenched by adding of a loading buffer (20% ficoll, 0.025% bromophenol blue, 0.025% xylene cyanol). The samples were loaded onto the running gel immediately after quenching the reaction with 1 min intervals. Formation of the complex miRNA/conjugate was analyzed by electrophoresis in 15% native PAAG at 4 °C. The gels were analyzed using Molecular Imager FX. The extent of binding of conjugate to miRNA was determined as a ratio of radioactivity measured in the complex to the total radioactivity applied onto the gel lane.

### 4.5. Ribonuclease Activity Assay

The reaction mixture (10 µL) contained 800 cpm (Cherenkov’s counting) of [^32^P]-miRNA, 1 μM unlabeled miRNA, one of the conjugates at a concentration 10 or 20 μM and one of the buffers: 50 mM Tris-HCl, pH 7.0, 200 mM KCl, 1 mM EDTA–buffer (1) or 20 mM Tris-HCl, pH 7.8, 40 mM KCl, 8 mM MgCl_2_, 1 mM DTT, 0.02 mg/mL BSA–buffer (2). The mixture was incubated at 37 °C for 0–72 h and quenched by precipitation of RNA with 2% LiClO_4_ in acetone (90 µL). RNA was collected by centrifugation and dissolved in loading buffer (8 M urea, 0.025% bromophenol blue, 0.025% xylene cyanol). RNA cleavage products were analyzed in 18% PAAG/8M urea using TBE (100 mM Tris-borate, pH 8.3, 2 mM EDTA) as running buffer. To identify cleavage sites, an imidazole and T1-ladders produced by partial RNA cleavage with 2 M imidazole buffer (pH 7.0) [67] and with RNase T1 [68], respectively, were run in parallel. To obtain quantitative data, gels were dried and analyzed using Molecular Imager FX. The total extent of RNA cleavage and the extent of RNA cleavage at each individual site were determined by using Quantity One software v.4.1 (Bio-Rad, Hercules, CA, USA).

### 4.6. Ribonuclease Activity Assay Using Dual Conjugates and RNase H

The reaction mixture (10 µL) containing 20 mM Tris-HCl, pH 7.8, 40 mM KCl, 8 mM MgCl_2_, 1 mM DTT, 0.02 mg/mL BSA, 800 cpm (Cherenkov’s counting) of [^32^P]-miRNA, 1 µM unlabeled miRNA, 20 µM either DC or Dual oligonucleotide was incubated at 37 °C for 20 min then RNase H (5 or 100 U/mL) was added and the mixture was further incubated for 48 h. Aliquots were taken at 0, 1, 6, 24 and 48 h then the reaction was quenched, RNA cleavage products were collected and analyzed as described above.

### 4.7. Ribonuclease Activity Assay at Different Concentrations of MgCl_2_

Reaction with different concentration of MgCl_2_ was performed in reaction mixtures (8 µL) containing 320 cpm (Cherenkov’s counting) of [^32^P]-miR-18a, 1 μM unlabeled miRNA, 20 µM of miR-18a-DC in buffers contained 100 mM KCl, 50 mM Tris-HCl pH 7.0, 1mM DTT, 0.02 mg/mL BSA and MgCl_2_ at concentration 0, 2, 4, 8 or 15 mM at 37 °C for 48 h. RNA cleavage products were collected and analyzed as described above.

### 4.8. Ribonuclease Activity Assay at Different Concentrations of KCl

Reaction with different concentration of KCl was performed in reaction mixtures (8 µL) containing 320 cpm (Cherenkov’s counting) of [^32^P]-miR-17, 1 μM unlabeled miRNA, 20 µM of miRNA-17-DC in buffers contained 20 mM Tris-HCl, pH 7.8, 8 mM MgCl_2_, 1 mM DTT, 0.02 mg/mL BSA, and KCl at concentration 0, 40, 100, 200 or 400 mM at 37 °C for 48 h. RNA cleavage products were collected and analyzed as described above.

### 4.9. pH Profile of Ribonuclease Activity of DCs

pH profile of miR-17 and miR-18a cleavage by the DCs was assayed at pH from 3.7 to 9.5. The buffers were: 50mM sodium acetate-CH3COOH for pH 3.7–5.0; 50mM MES-HCl for pH 4.5–6.0; 50 mM bis-tris-propane-KOH for pH 6.0–9.5; and 50 mM Tris-HCl for pH 7.0–9.0. All buffers contained 100 mM KCl, 1 mM DTT, 0.02 mg/mL BSA and 2 mM MgCl_2_. Reactions were performed in reaction mixtures (8 µL) containing 320 cpm (Cherenkov’s counting) of [^32^P]-miRNA, 1 μM unlabeled miRNA, 10 μM miRNA-17-DC or 20 µM miR-18a-DC, one of the buffers at 37 °C for 48 h. RNA cleavage products were collected and analyzed as described above.

### 4.10. Stability of DCs and ONs in 10% FBS

Stability of miRNA-21-DC and miRNA-155-DC and corresponding ONs was carried out in reaction mixtures (90 µL) containing DCs or ONs at a concentration of 0.1 mg/mL in Dulbecco’s Modified Eagle Medium (DMEM) with 10% FBS at 37 °C for 24 h. Aliquots (10 μL) were taken after 0, 0.5, 1, 2, 4, 8, and 24 h of incubation. The reaction was stopped by adding 10 μL of 8 M urea, and the reaction mixture was immediately frozen in liquid nitrogen. DCs and ONs cleavage products were analyzed in 12% PAAG/8M urea. To identified products, gels were stained with the Stains-All dye and photographed using a gel documentation system.

### 4.11. Statistics

Data are presented as mean with standard error (mean ± s.e.) from three independent experiments. Data were statistically processed using one-way ANOVA using post-hoc Tukey test. *p* < 0.05 was considered to be statistically significant.

## 5. Conclusions

We have developed new miRNA-targeted artificial ribonucleases–Dual miRNases, the optimal functioning conditions of which are close to a physiological intracellular environment. miRNA degradation by Dual miRNases significantly increases in the presence of RNase H, which undoubtedly may contribute to the efficient irreversible inactivation of tumor-associated miRNAs in vitro and in vivo. Due to the increased activity at lowered pH, Dual miRNases could be beneficial in acidic tumor conditions representing efficient and tumor-selective therapy, avoiding side effects and systemic toxicity, which is in a high demand nowadays.

## Figures and Tables

**Table 1:** Conjugates and oligonucleotides used in the study.

Conjugate	Sequence 5′→3′
	**DC**
miRNA-21-DC	TCAACATC–linker 1–[GRLRL]_2_–linker 2–GATAAGCTA
miRNA-18a-DC	TATCTGC–linker 1–[GRLRL]_2_–linker 2–ATGCACCTTA
miRNA-17-DC	CTACCTGC–linker 1–[GRLRL]_2_–linker 2–AAGCACTTTG
miRNA-155-DC	ACCCCTAT–linker 1–[GRLRL]_2_–linker 2–TTAGCATTAA
	**SC**
miRNA-21-SC	[GRLRL]_2_–linker 2–GATAAGCTA
miRNA-18a-SC	[GRLRL]_2_–linker 2–ATGCACCTTA
miRNA-17-SC	[GRLRL]_2_–linker 2–AAGCACTTTG
miRNA-155-SC	[GRLRL]_2_–linker 2–TTAGCATTAA
	**ON**
miRNA-21-ON	TCAACATC–TEG–TEG–GATAAGCTA
miRNA-18a-ON	TATCTGC–TEG–TEG–ATGCACCTTA
miRNA-17-ON	CTACCTGC–TEG–TEG–AAGCACTTTG
miRNA-155-ON	ACCCCTAT–TEG–TEG–TTAGCATTAA

Linker 1–aminohexyl linker, linker 2–thiohexyl linker, TEG—triethylene glycol linker. SC—Single oligonucleotide-peptide conjugate (Single conjugate), DC—Dual oligonucleotide-peptide conjugate (Dual conjugate). ON—Dual oligonucleotide. Underlined base–2-aminoadenine. Please note that in the case of DC and SC, the peptide sequence is written in the direction: C–terminus → N–terminus.

## Supplementary Materials

The following are available online at https://www.mdpi.com/1420-3049/25/10/2459/s1, Table S1, Role of targeted miRs in oncopathologies; Figure S1, The structure of the pair 2-aminoadenine – uracil; Figure S2, IEX HPLC of SCs and DCs; Figure S3, Reverse phase HPLC of DCs; Figure S4, Urea-PAGE analysis of SCs and DCs; Figures S5–S8, ^1^H-NMR spectroscopy of SCs and DCs, Figure S9, Hybridization of dual ONs with miRNAs; Table S2, miRNA cleavage by DCs in buffer (1) and (2); Tables S3 and S4, Influence of Mg^2+^ and K^+^ ions on cleavage by DCs; Figure S10, Self-cleavage of miR-17 at different pH; Figure S11, Influence of BSA on miRNA cleavage by DC; Table S5, Sequences of miRNAs; Figure S12, Stability of DCs and dual ONs in 10% FBS.
