# Supplementary material for: Dual miRNases for Triple Incision of miRNA Target: Design Concept and Catalytic Performance
Source: Molecules. 2020 May 25;25(10):2459. doi: 10.3390/molecules25102459 (PMC7287882; doi:10.3390/molecules25102459)
Supplement: Supplementary file 1 [file molecules-25-02459-s001.pdf]

SUPPLEMENTARY MATERIALS

Dual miRNases for triple incision of miRNA target: design concept and catalytic performance

Olga A. Patutina [1], Daria A. Chiglintseva [1], Elena V. Bichenkova [2], Svetlana K. Gaponova [1], Nadezhda L. Mironova [1], Valentin V. Vlassov [1] and Marina A. Zenkova [1,*]

[1] Laboratory of Nucleic Acids Biochemistry, Institute of Chemical Biology and Fundamental Medicine SB RAS, Lavrentiev's ave. 8, 630090, Novosibirsk, Russia
[2] School of Health Sciences, Faculty of Biology, Medicine and Health, University of Manchester, Oxford Rd, Manchester, M13 9PT, UK

* Correspondence: marzen@niboch.nsc.ru; Tel.: +7-383-363-51-60

Table S1. The role of miR-21, miR-155, miR-17 and miR-18a in oncological diseases.

miRNA	mRNA targets	Cellular function under control	Malignancy	Ref.
miR-21	PDCD4, TPM1, PTEN, SPRY1, SPRY2, MARCKS	Proliferation, differentiation, migration, invasion, angiogenesis, apoptosis	Breast, non-small cellular lung cancer, lymphosarcoma, melanoma, gastric and colorectal cancer	[4,14,15]
miR-155	GABRA1, FOXO3a, BACH1, VHL	Proliferation, migration, invasion, angiogenesis	B-cell lymphoma, hepatocellular carcinoma, lung and breast cancer	[4,16,20]
miR-17	Transcription factors, matrix metalloproteinases	Proliferation, migration, apoptosis	Colorectal, gastric and breast cancer	[4,17]
miR-18a	Proteins of phosphatidylinositol 3-kinase-protein kinase B (PI3K/AKT), MEK/extracellular signal-regulated kinase (ERK), mTOR and Wnt/β-catenin pathways	Proliferation, migration, invasion	Nasopharyngeal carcinoma, clear cell renal cell carcinoma, cervical cancer	[4,18,19]

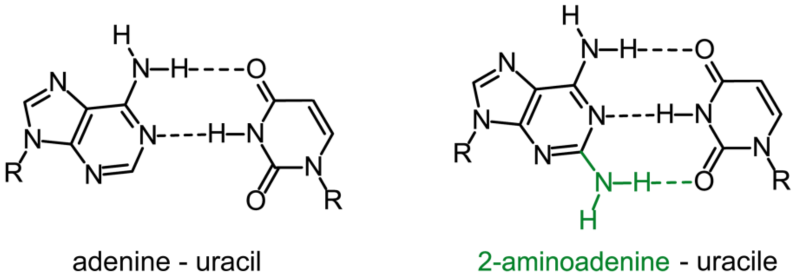

Figure S1. The structure of the pair adenine-uracile and 2-aminoadenine-uracil.

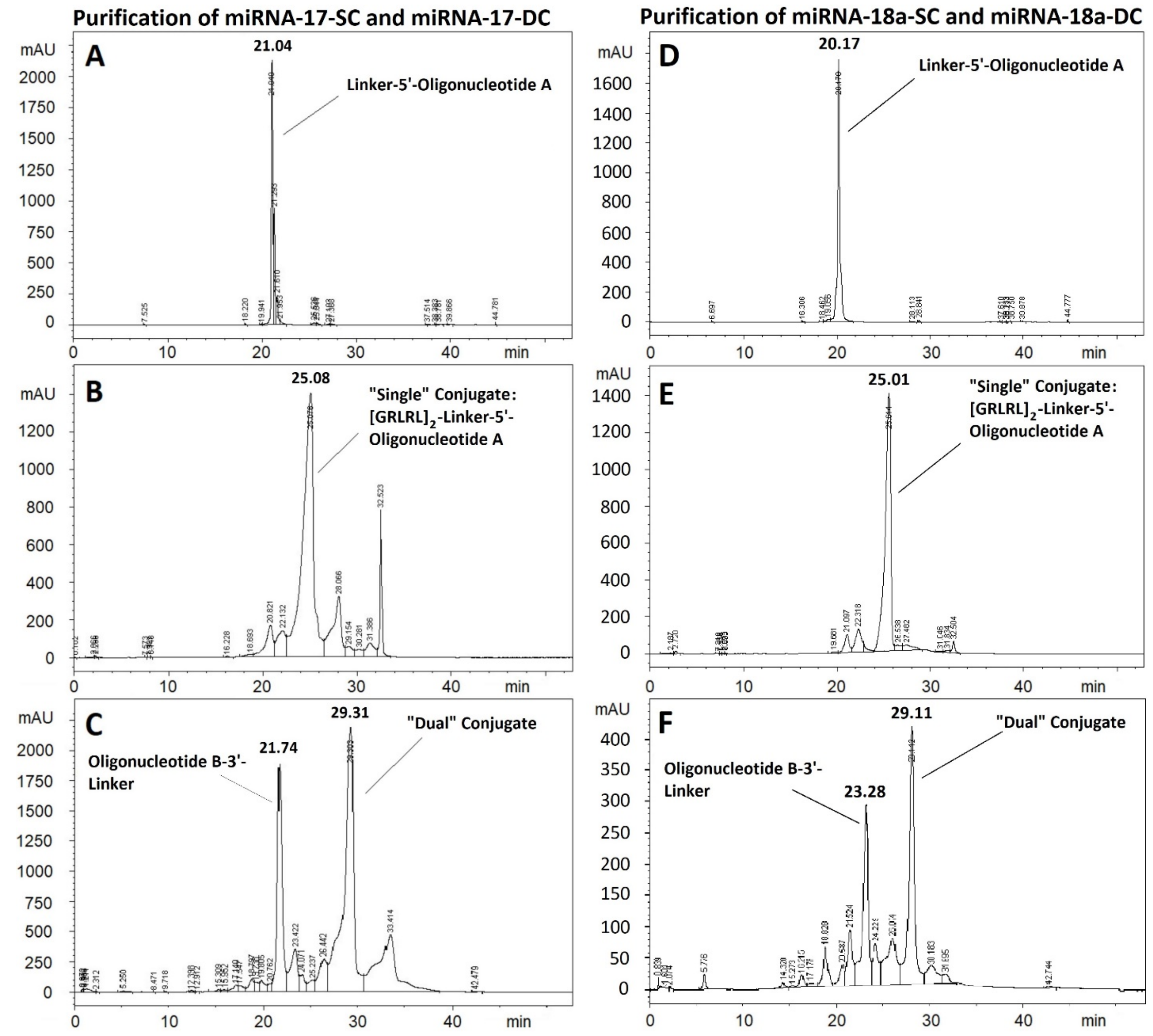

Figure S2. Representative examples of IEX HPLC showing purification of the Single and Dual conjugates after the first and second coupling reactions, respectively, as a part of the synthetic route of miRNA-17-DC (left; **A** – **C**) and miRNA-18a-DC (right; **D** – **F**). (**A** and **D**) Starting Oligonucleotide A bearing 5'-thiohexyl linker with the retention time of 21.04 or 20.17 min, respectively. (**B** and **E**) Single conjugate (miRNA-17-SC or miRNA-18a-SC, respectively) after the first coupling step, showing the shift in the retention time by 4 or 5 min as compared to unmodified Oligonucleotide A. (**C** and **F**) Dual conjugate (miRNA-17-DC or miRNA-18a-DC, respectively) after the second coupling step, showing the additional 4-minute shift in the retention time as compared to that of the Single conjugate. IEX was performed using a Clarity Oligo-WAX column. The mobile phase included 10 mM Tris-Cl (pH 7.25) and 10% AcCN in water as eluent A, and 2 M NaCl, 10 mM Tris-Cl (pH 7.25) and 10% AcCN in water as eluent B. The following gradient was applied: 100% A for 5 min, followed by 0% B to 100% B in 32 min. The flow rate was maintained at 2.0 mL/min, and the UV absorbance was monitored at 260 nm. Similar shifts in the retention times were witnessed during IEX HPLC purification of the other conjugates.

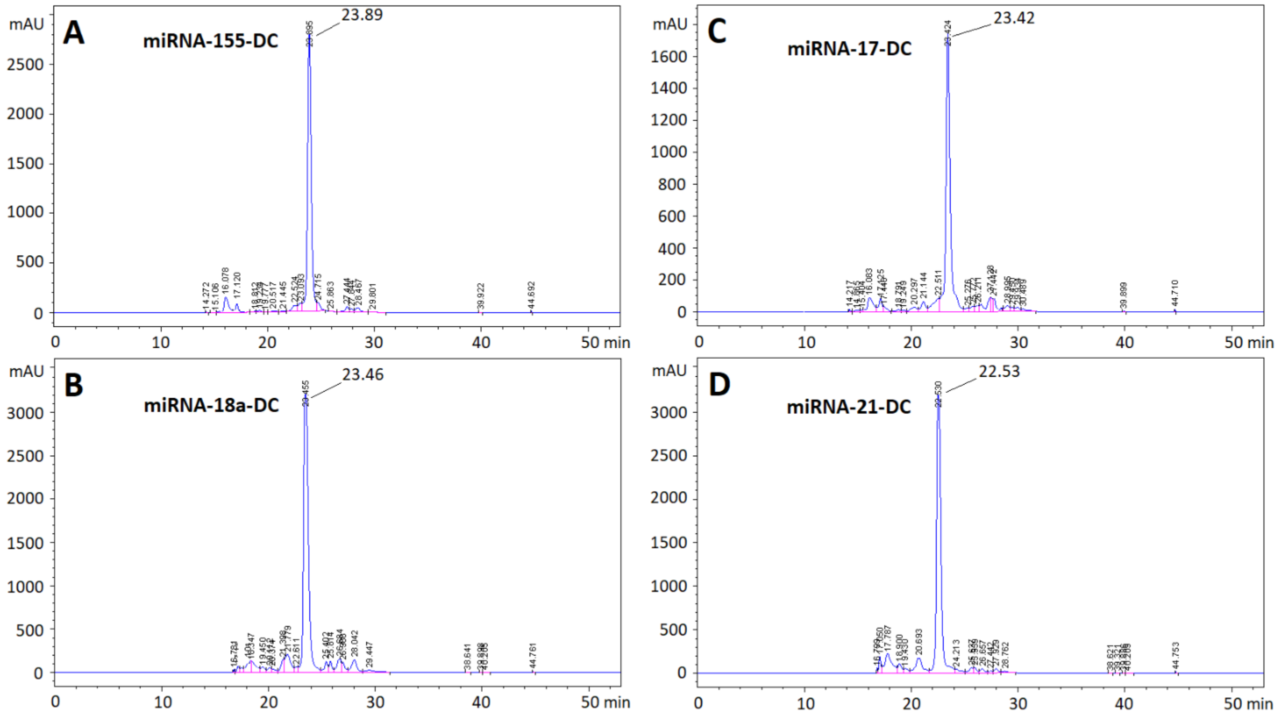

Figure S3. Reversed phase HPLC purification of the Dual conjugates miRNA-155-DC (**A**), miRNA-18a-DC (**B**), miRNA-17-DC (**C**) and miRNA-21-DC (**D**). RP-HPLC was carried out using a semi-preparative column Luna C18 (Phenomenex; CA, USA). The mobile phase included 0.05 M $LiClO_4$ in water as eluent A and 0.05 M $LiClO_4$ in AcCN as eluent B. The absorbance was monitored at 260 nm, and the following gradient was applied: 100% A for 3 min, 0% B to 100% B in 30 min, followed by washing with 100% B over 10 min and with 100% A over the next 20 min. The flow rate was maintained at 2.0 mL/min. The Single conjugates miRNA-155-SC, miRNA-18a-SC, miRNA-17-SC and miRNA-21-SC were purified by RP-HPLC in a similar way.

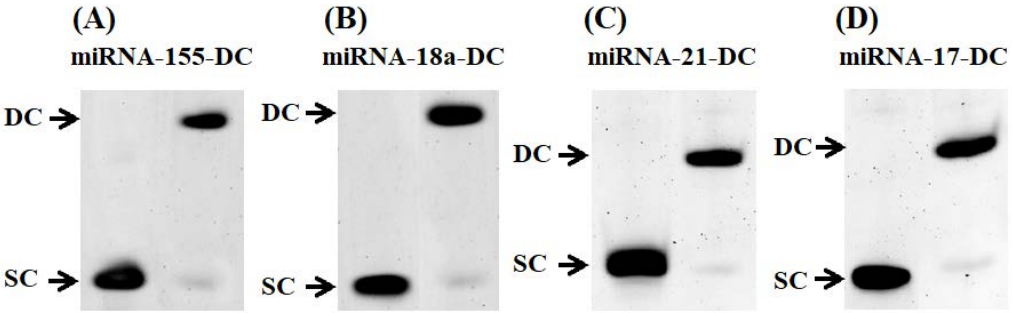

PAGE	Dual Conjugate	Purity (%)
**(A)**	miRNA-155-DC	**95.2%**
**(B)**	miRNA-18a-DC	**95.6%**
**(C)**	miRNA-21-DC	**96.5%**
**(D)**	miRNA-17-DC	**95.6%**

Figure S4: Urea-PAGE (20% PAA/8 M Urea) analysis showing homogeneity of the Dual conjugates (DC) miRNA-155-DC (**A**), miRNA-18a-DC (**B**), miRNA-21-DC (**C**) and miRNA-17-DC (**D**) isolated as the main fractions between 22 and 25 min of the reversed phase HPLC purification (see **Figure S3**). Urea-PAGE analysis shows the reduced electrophoretic mobility of the Dual conjugates as compared to that for the corresponding Single conjugates (**SC**) miRNA-155-SC, miRNA-18a-SC, miRNA-21-SC and miRNA-17-SC, respectively.

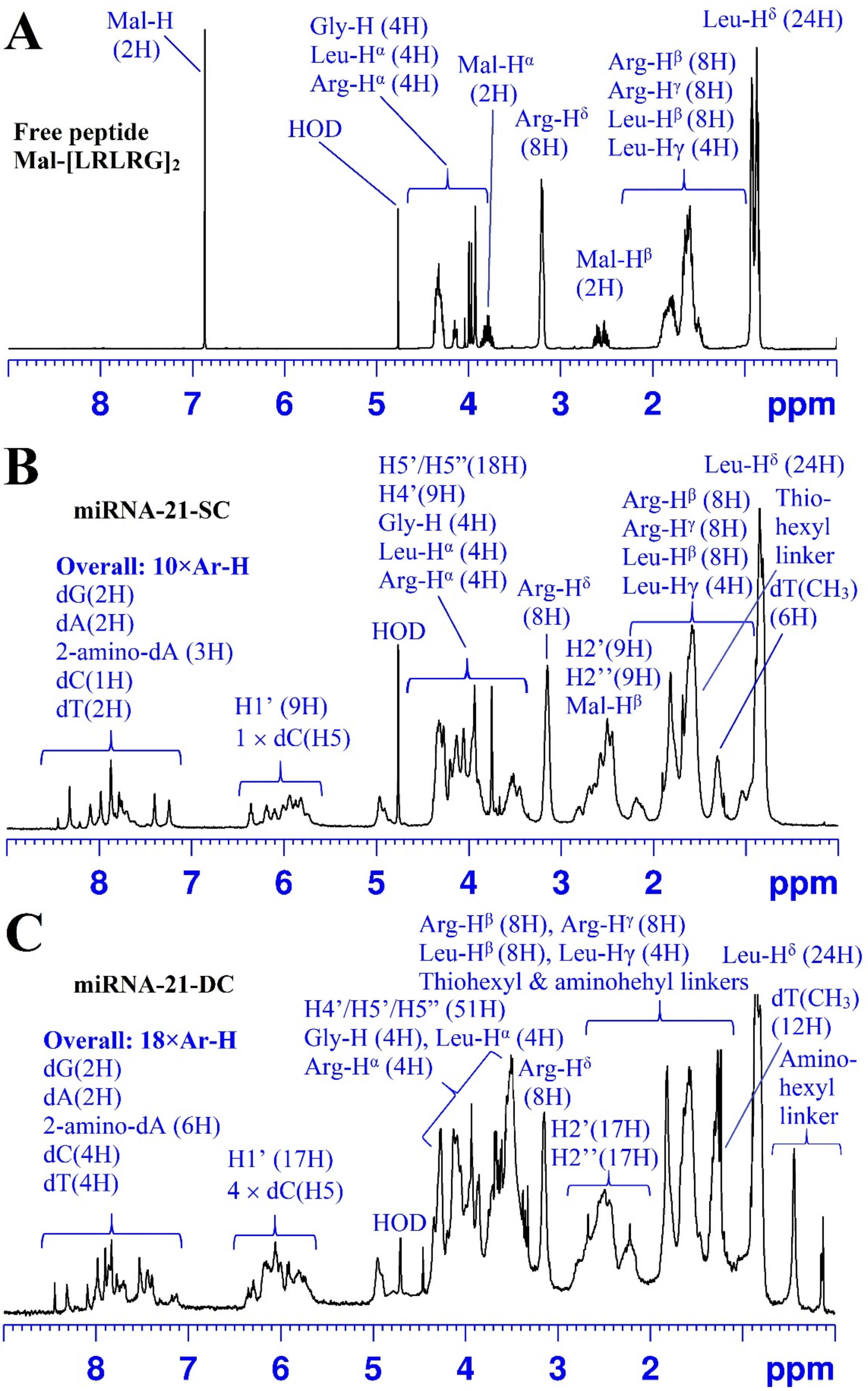

Figure S5. *Step-by-step* characterization of the Single and Dual peptidyl-oligonucleotide conjugates produced for targeting miR-21 sequence using ^{1}H-NMR spectroscopy. NMR spectra of the unconjugated **Mal-[LRLRG]₂** peptide (**A**), intermediate Single conjugate **miRNA-21-SC** (**B**) and Dual conjugate **miRNA-21-DC** (**C**). Spectra were recorded in D_2O at 25 °C using 400 MHz, Bruker Avance II+ 400 NMR spectrometer.

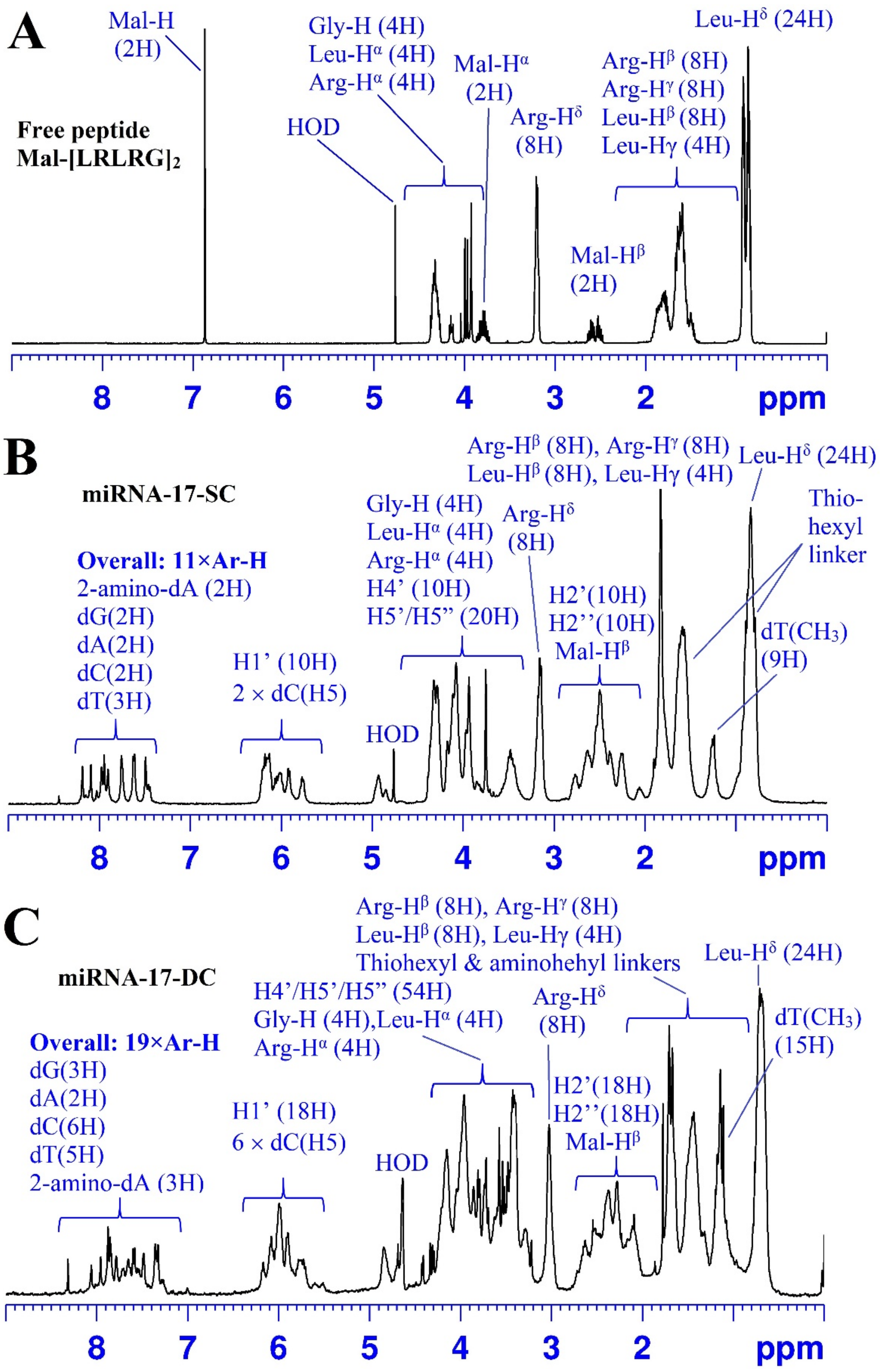

Figure S6. *Step-by-step* characterization of the Single and Dual peptidyl-oligonucleotide conjugates produced for targeting miR-17 sequence using ^{1}H-NMR spectroscopy. NMR spectra of the unconjugated **Mal-[LRLRG]$_2$** peptide (**A**), intermediate Single conjugate **miRNA-17-SC** (**B**) and Dual conjugate **miRNA-17-DC** (**C**). Spectra were recorded in D_2O at 25 °C using 400 MHz, Bruker Avance II+ 400 NMR spectrometer.

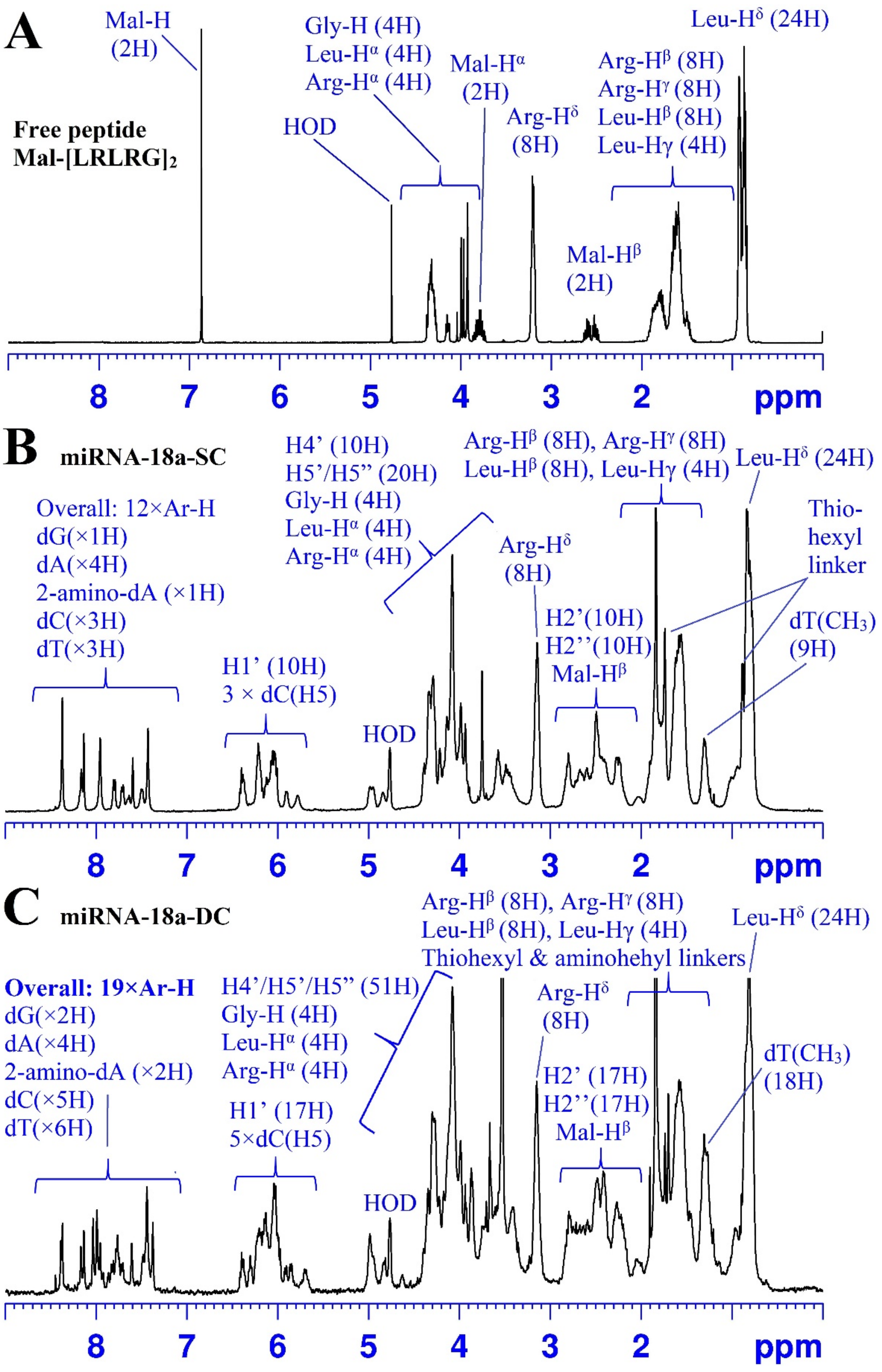

Figure S7. *Step-by-step* characterization of the Single and Dual peptidyl-oligonucleotide conjugates produced for targeting miR-18a sequence using ^{1}H-NMR spectroscopy. NMR spectra of the unconjugated **Mal-[LRLRG]$_2$** peptide (**A**), intermediate Single conjugate **miRNA-18a-SC** (**B**) and Dual conjugate **miRNA-18a-DC** (**C**). Spectra were recorded in D_2O at 25 °C using 400 MHz, Bruker Avance II+ 400 NMR spectrometer.

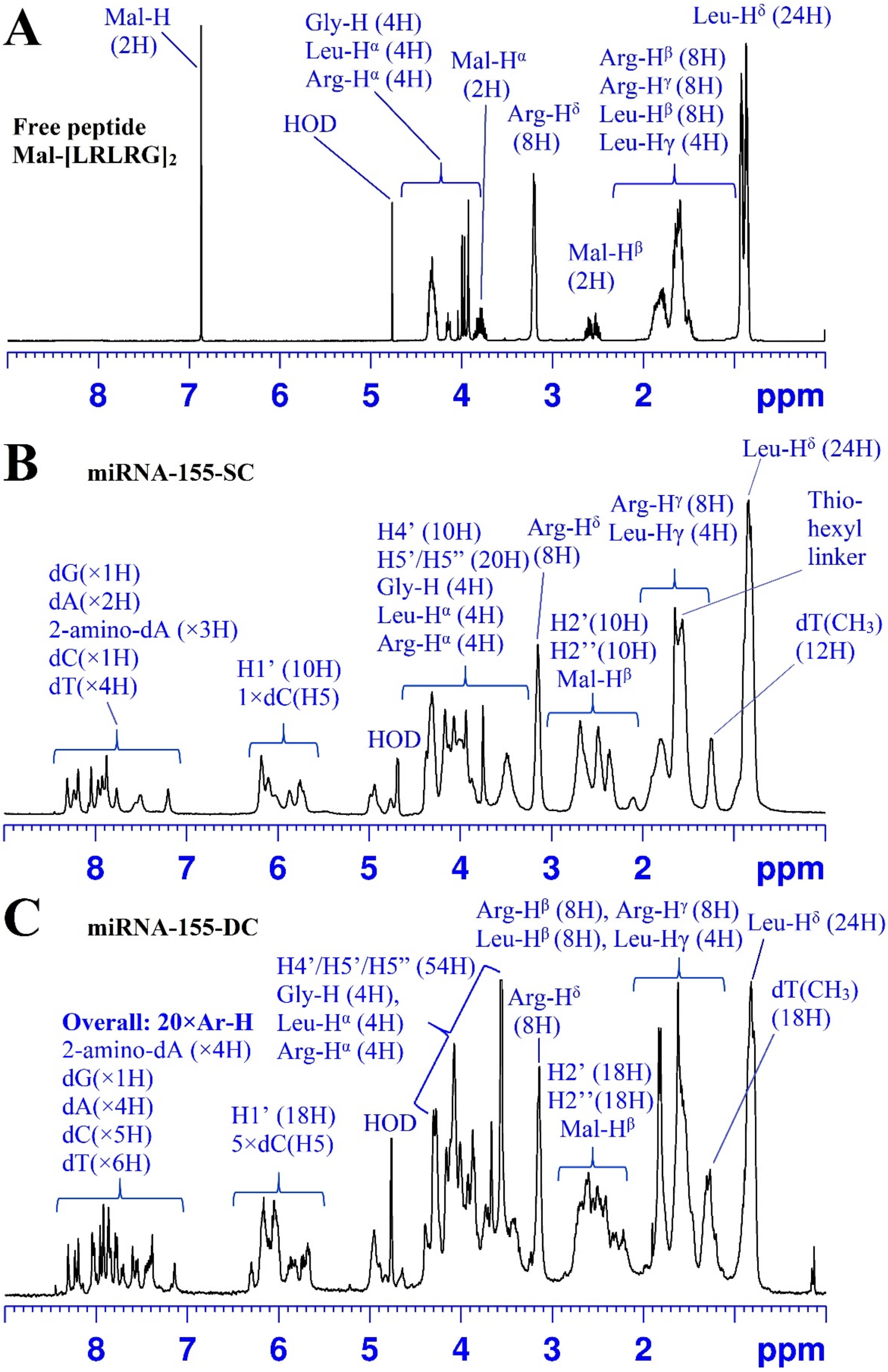

Figure S8. *Step-by-step* characterization of the Single and Dual peptidyl-oligonucleotide conjugates produced for targeting miR-155 sequence using ^{1}H-NMR spectroscopy. NMR spectra of the unconjugated **Mal-[LRLRG]$_2$** peptide (**A**), intermediate Single conjugate **miRNA-155-SC** (**B**) and **miRNA-155-DC** (**C**). Spectra were recorded in D_2O at 25 °C using 400 MHz, Dual conjugate Bruker Avance II+ 400 NMR spectrometer.

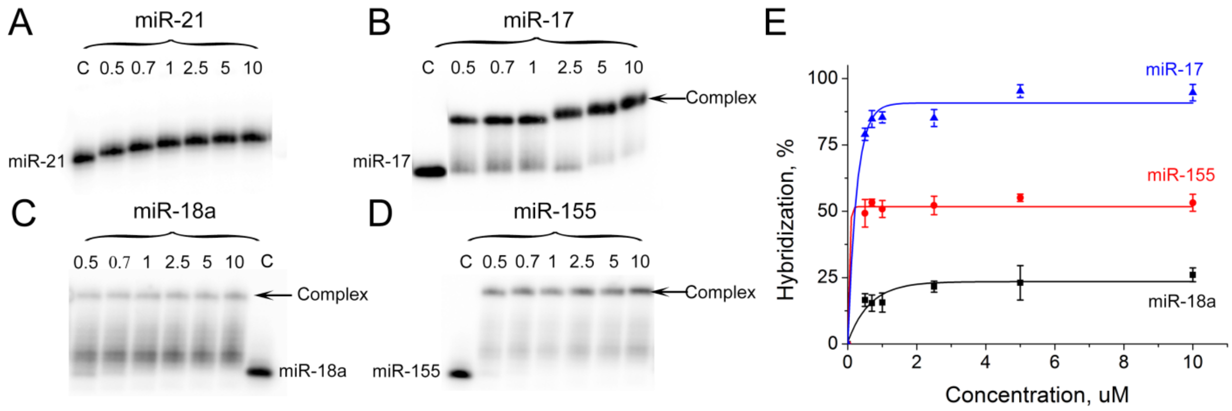

Figure S9. Hybridization of 5'-[^{32}P]-labeled miRNAs with the Dual oligonucleotides (ONs) miRNA-21-ON, miRNA-17-ON, miRNA-18a-ON and miRNA-155-ON. **A, B, C** and **D.** Radioautographs of 15% native PAAG, showing hybridization of the corresponding ONs with miR-21, miR-17, miR-18a and miR-155, respectively. miRNAs (1 μM) were incubated with ONs (0.1 – 10 μM) in buffer (1), containing 50 mM Tris-HCl, pH 7.0, 200 mM KCl and 1 mM EDTA at 37 °C for 45 min. The samples were loaded onto the running gel immediately after quenching the reaction with 1 min intervals. The concentration (μM) of ONs is indicated on the top of electrophoregrams. **E.** Concentration dependencies of ONs hybridization with miR-17, miR-18a and miR-155. Data are presented as mean ± s.e.

Table S2. Efficiency of miRNA cleavage and observed rate constants (K_{obs}) of miRNA-targeted DCs in buffer (1) and (2).

	**Total Cleavage, %**								
	**miR-17**		**miR-18a**		**miR-21**	**miR-21_1**		**miR-155**	
**Time, h**	***buff 1***	***buff 2***	***buff 1***	***buff 2***	***buff 2***	***buff 1***	***buff 2***	***buff 1***	***buff 2***
**2**	0	1,5	0	1,4	4	0,7	-	0	15
**6**	0	4	1	5	13	-	14	0	41
**24**	1	18	3	19	30	10	48	0,5	56
**48**	2	32	7	23	30	18	63	16	57
**72**	5	40	8	37	34	-	-	21	72
**K_{obs}, $\times 10^{-6}$, s^{-1}**	18.0± 1.6	190.0± 4.5	34.2± 2.4	222.0± 6.1	328.0± 32.9	107.0± 4.3	373.0± 55.9	85.2± 17.0	543.0± 173.0

Table S3. Cleavage of 5'-[^{32}P]-labeled miR-18a by DC in buffer, containing different concentration of $MgCl_2$.

**Mg^{2+}, mM**	**0**	**2**	**4**	**8**	**15**
Cleavage, %	26.4±4.0	28.8±3.9	24.3±2.7	23.1±3.1	27.2±4.3

miRNA [1 µM] and DC [20 µM] were incubated at 37°C for 48 h. Buffer: 50 mM Tris-HCl, pH 7.0, 100 mM KCl, 1 mM DTT, 0.02 mg/mL BSA and $MgCl_2$ at a concentration range from 0 to 15 mM.

Table S4. Cleavage of 5'-[^{32}P]-labeled miR-17 by DC in buffer, containing different concentration of KCl.

**K^+, mM**	**0**	**40**	**100**	**200**	**400**
Cleavage, %	13.2±5.0	22.5±4.3	26.4±2.0	13.7±3.5	14.1±3.8

miRNA [1 µM] and DC [20 µM] were incubated at 37°C for 48 h. Buffer: Buffer: 20 mM Tris-HCl, pH 7.8, 8 mM $MgCl_2$, 1 mM DTT, 0.02 mg/mL BSA and KCl at a concentration range from 0 to 400 mM.

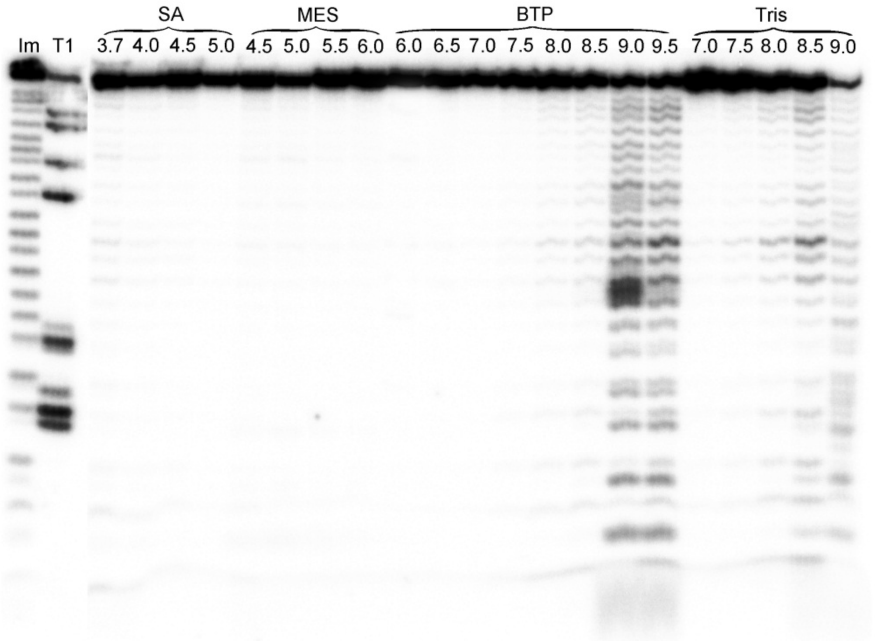

Figure S10. Self-cleavage of 5'-[^{32}P]-labeled miR-17 at different pH. Buffers are listed in the "Materials and methods" section. Radioautograph of 18% denaturing PAAG showing the cleavage products of miR-17. miRNA-17 at 1 µM concentration was incubated at 37°C for 48 h. Lanes Im and T1 – imidazole ladder and partial RNA digestion with RNase T1, respectively.

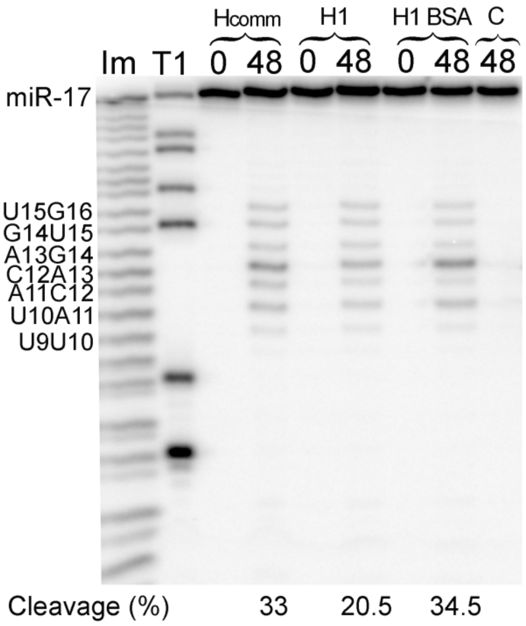

Figure S11. Cleavage of 5'-[^{32}P]-labeled miR-17 by DC in three buffers: Hcomm – commercial RNase H buffer; H1 – buffer, containing 20 mM Tris-HCl, pH 7.8, 40 mM KCl, 8 mM $MgCl_2$, 1 mM DTT, and H1 BSA – buffer H1 with addition of 0.02 mg/mL BSA. Radioautograph of 18% denaturing PAAG, showing the cleavage products of miR-17. miRNA (1 µM) and DC (20 µM) were incubated at 37°C for 48 h. Lanes Im and T1 – imidazole ladder and partial RNA digestion with RNase T1, respectively; C – miRNA was incubated in the absence of the conjugate in buffer H1. The incubation time (in hours) is indicated at the top.

Table S5. Sequences of miRNAs used in the study.

miRNA	Sequence 5’-3’
miRNA-21	UAGCUUAUCAGACUGAUGUUGA
miR-21_1	UAGCUUAUCAUACAGAUGUUGA
miRNA-17	CAAAGUGCUUACAGUGCAGGUAG
miRNA-18a	UAAGGUGCAUCUAGUGCAGAUA
miRNA-155	UUAAUGCUAAUUGUGAUAGGGGU

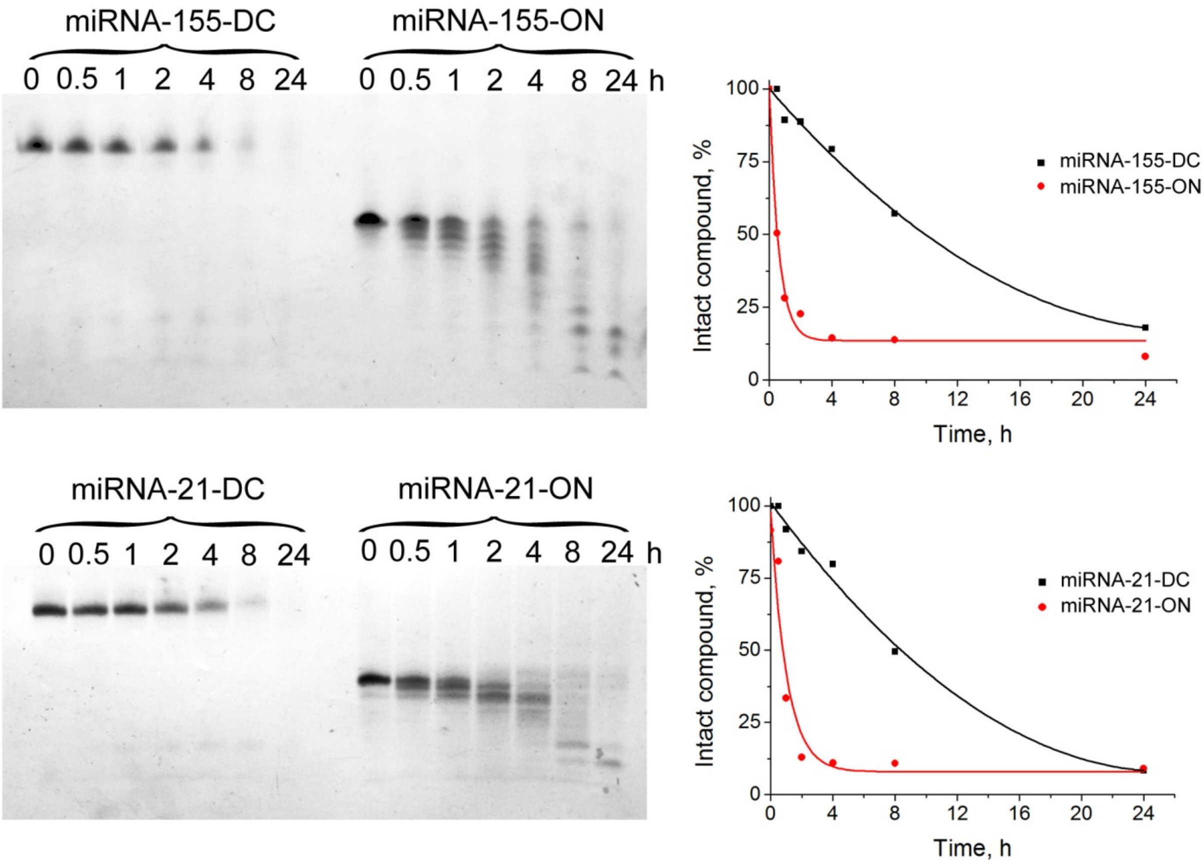

Figure S12. Stability of Dual conjugates (DCs) and corresponding Dual oligonucleotides (ONs) targeted to miR-155 and miR-21 in cell medium DMEM supplemented with 10% FBS. Images of the 12% PAAG/8M urea, stained with Stains-All and kinetics of degradation of DCs and ONs. Conjugates and oligonucleotides at a concentration of 0.1 mg/mL were incubated in DMEM supplemented with 10% FBS at 37 °C for 24 h.
